# Transcriptional and epigenetic modulation of autophagy promotes EBV oncoprotein EBNA3C induced B-cell survival

**DOI:** 10.1038/s41419-018-0668-9

**Published:** 2018-05-22

**Authors:** Shaoni Bhattacharjee, Priyanka Bose, Krishna Patel, Shatadru Ghosh Roy, Chandrima Gain, Harsha Gowda, Erle S. Robertson, Abhik Saha

**Affiliations:** 1 Department of Life Sciences, Presidency University, 86/1 College Street, Kolkata, 700073 West Bengal India; 2 Institute of Bioinformatics, Discoverer Building, International Tech Park, Whitefield, Bangalore 560066 India; 3 Department of Otorhinolaryngology-Head and neck surgery, and the Tumor Virology Program, Abramson Comprehensive Cancer Center, Perelman School of Medicine at the University of Pennsylvania, Philadelphia, Pennsylvania PA-19104 USA

## Abstract

Epstein-Barr virus (EBV) oncoprotein EBNA3C is indispensable for primary B-cell transformation and maintenance of lymphoblastoid cells outgrowth. EBNA3C usurps two putative cellular pathways—cell-cycle and apoptosis, essentially through modulating ubiquitin-mediated protein-degradation or gene transcription. In cancer cells, these two pathways are interconnected with autophagy,—a survival-promoting catabolic network in which cytoplasmic material including mis/un-folded protein aggregates and damaged organelles along with intracellular pathogens are degraded and recycled in lysosomal compartments. Studies have shown that tumor viruses including EBV can manipulate autophagy as a survival strategy. Here, we demonstrate that EBNA3C elevates autophagy, which serves as a prerequisite for apoptotic inhibition and maintenance of cell growth. Using PCR based micro-array we show that EBNA3C globally accelerates autophagy gene transcription under growth limiting conditions. Reanalyzing the ENCODE ChIP-sequencing data (GSE52632 and GSE26386) followed by ChIP-PCR demonstrate that EBNA3C recruits several histone activation epigenetic marks (H3K4me1, H3K4me3, H3K9ac, and H3K27ac) for transcriptional activation of autophagy genes, notably *ATG3*, *ATG5*, and *ATG7* responsible for autophagosome formation. Moreover, under growth limiting conditions EBNA3C further stimulates the autophagic response through upregulation of a number of tumor suppressor genes, notably cyclin-dependent kinase inhibitors—*CDKN1B* (p27^Kip1^) and *CDKN2A* (p16^INK4a^) and autophagy mediated cell-death modulators—*DRAM1* and *DAPK1*. Together our data highlight a new role of an essential EBV oncoprotein in regulating autophagy cascade as a survival mechanism and offer novel-targets for potential therapeutic expansion against EBV induced B-cell lymphomas.

## Introduction

Epstein-Barr virus (EBV) is a lymphotropic gammaherpesvirus that asymptomatically persists in >95% of the world population^1^. However, in immuno-compromized individuals, the loss of immune-control of the latently-infected B-cells results in the emergence of several B-cell lymphomas^2–4^. In vitro, EBV can efficiently transform resting B-lymphocytes into lymphoblastoid cell-lines (LCLs), providing an excellent model to study EBV-induced B-cell transformation and B-cell lymphomagenesis^5^. In LCLs, EBV expresses six nuclear antigens (EBNAs) and three membrane proteins (LMPs) along with several non-coding RNAs^1,6^. Of these, five viral latent proteins including EBNA2, EBNA3A, EBNA3C, EBNALP, and LMP1 are indispensible for B-cell transformation and subsequent lymphoma development^6,7^.

EBNA3C interferes with a number of cellular pathways—most strikingly cell-cycle/apoptosis and ubiquitin-targeted protein-degradation machineries either through direct protein–protein interaction or transcriptional regulation^7,8^. EBNA3C functions as a strong transcription factor affecting both viral and cellular gene expressions through interacting with a plethora of cellular transcription factors, epigenetic, and post-translational modifiers^9–28^. A number of these EBNA3C deregulated cellular components can also influence autophagy pathway. However, whether EBNA3C is directly involved in deregulating the autophagy machinery to promote its oncogenic property remains unanswered.

Autophagy is an evolutionarily conserved cell mechanism that sequesters cytoplasmic materials such as damaged organelles, protein aggregates, and intracellular pathogens for degradation in lysosomal compartments^2–4^. Autophagy can act as both cyto-protective and cell-death mechanism depending on various extra-cellular stimuli^2^. In response to nutrient deprivation, a model that reflects cancer micro-environment, cells undergo autophagy activation selectively targeting various signalling cascades besides proteolytic clearance of protein aggregates and damaged organelles, which essentially maintains the energy threshold and fundamental building blocks required for cancer cell outgrowth^2,3^.

Given the ability of autophagy to regulate diverse cellular processes, it is not surprising that tumor-viruses including EBV have evolved multiple strategies to interact with this pathway to promote their own survival^29–31^. Autophagy inhibition using specific inhibitors was shown to promote EBV lytic replication and may therefore affect its oncogenesis^32^. Current knowledge regarding the implications of autophagy in latently infected B-lymphocytes is primarily limited to two EBV latent antigens—EBNA1 and LMP1^30^. While LMP1 regulates autophagy to control its own turnover^33^, EBNA1-fragments but not EBNA3C and EBNA2 presented through MHC class-II via autophagy-lysosomal process^34,35^. It has also been suggested that EBNA1 accumulates in autophagosomes by blocking lysosomal acidification leading to a significant reduction in presenting EBNA1-fragments for CD4+ T-lymphocytes recognition^34^. Overall, these findings clearly indicate that EBV latent-antigens modulate autophagy and subsequently affect B-cell lymphomagenesis.

In this study, we have investigated the potential role of EBNA3C in regulating the autophagy pathway. Our results demonstrated that EBNA3C independently promoted autophagy as a pro-survival mechanism. EBNA3C expressing B-cells are more resistant to cell-death induced by autophagy inhibitors. EBNA3C increases the basal level of autophagy through transcriptional activation of several autophagy related genes (ATGs), which is further elevated in response to serum/amino acid starvation. Genome-wide ChIP-sequencing results (GSE52632^14^ and GSE26386^36^) on LCLs followed by ChIP-PCR data using EBNA3C expressing B-cells with or without autophagic induction clearly demonstrate that EBNA3C engages histone activation epigenetic marks to increase transcription of a number of autophagy modulators, essential for autophagosome biogenesis (*ATG3*, *ATG5*, *ATG7*) and cell-death mechanisms (*CDKN1B*, *CDKN2A*, *DRAM1,* and *DAPK1*). EBNA3C mediated autophagy deregulation can therefore be a strong avenue for targeted future pharmacological exploitation against multiple EBV-associated B-cell lymphomas particularly those generated in an immune-deficient background.

## Results

### EBNA3C-mediated cell-cycle activation and apoptotic-inhibition may be directly linked to autophagy

EBNA3C primarily targets two major signaling pathways—cell-cycle and apoptosis either by modulating gene-transcription or targeting ubiquitin-proteasomal pathway for regulating protein turn-over^7,8^. Many of these EBNA3C-interacting cellular partners can directly regulate the autophagy pathway. For example, the cyclin D1-pRb-E2F1 cascade was shown to influence autophagy gene transcriptions^37^. EBNA3C can also override autophagosome-mediated degradation in virus infected B-lymphocytes for antigen presentation^35^. However, the underlying mechanism was not described and led to a basic question—how does EBNA3C regulate the autophagy process and if it has a direct impact on EBNA3C mediated oncogenecity.

**Fig. 1:**
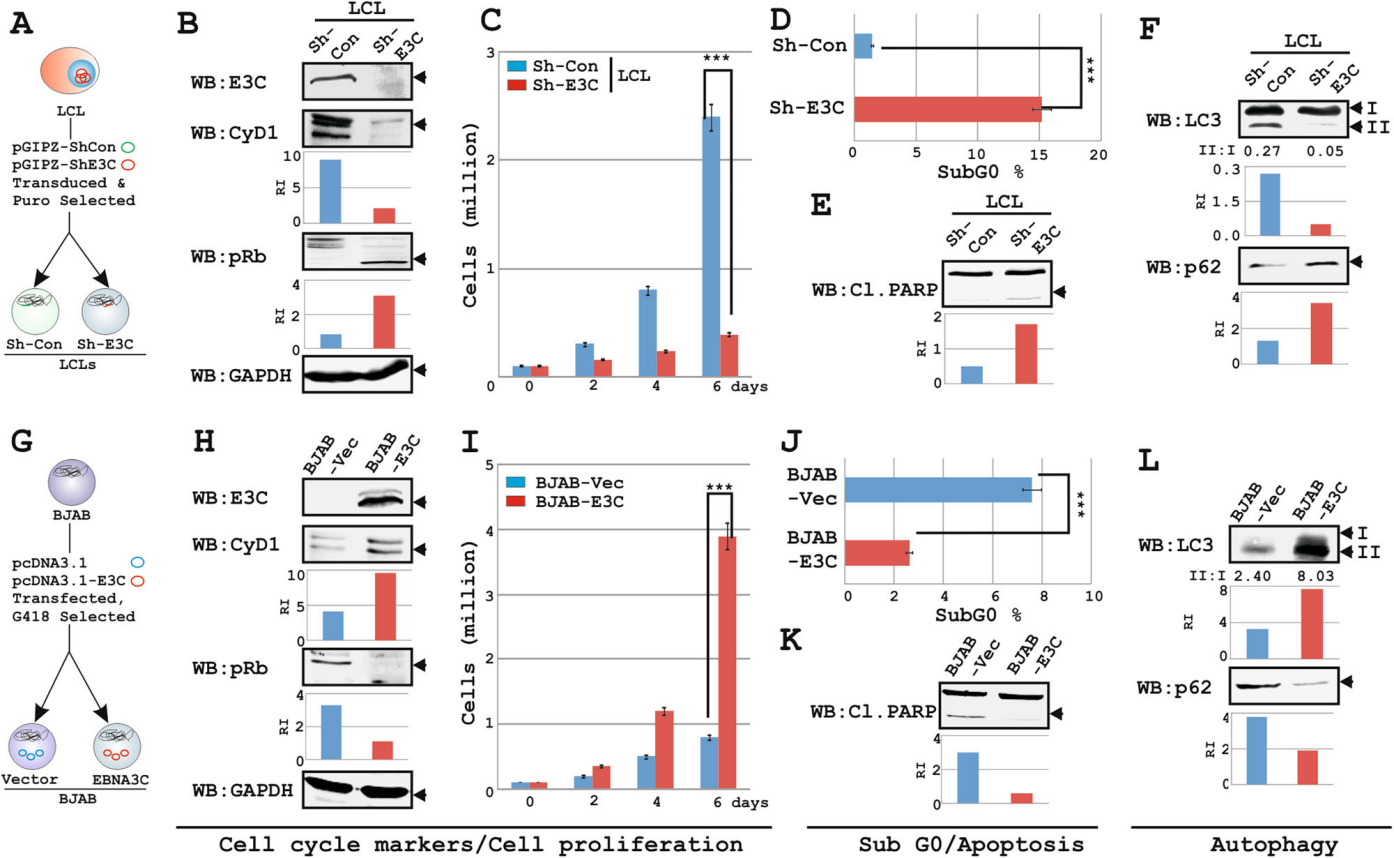
EBNA3C links autophagy to increase cell proliferation and inhibit apoptosis. **a** LCLs expressing either sh-control (Sh-Con) or sh-EBNA3C (Sh-E3C) and **g** BJAB-vector and BJAB stably expressing EBNA3C cells were subjected for (**b**, **e**, **f** and **h**, **k**, **l**) western blot (WB) analyses with indicated antibodies, (**c**, **i**) proliferation assay and (**d**, **j**) flow cytometry analyses. **b**, **e**, **f** and **h**, **k**, **l** For WB analyses ~10 × 10^6^ cells grown in complete RPMI were harvested, lysed in 1 × RIPA buffer and resolved in 9% and 13% SDS-PAGE, subsequently probed with indicated antibodies. WBs were done on single sample and the respective bands were cropped using software provided by Odyssey CLx Imaging System. Representative gel pictures are shown of two independent experiments. GAPDH was used as a loading control. **c**, **i** For proliferation assay ~0.1 × 10^6^ cells plated into each well of a six-well plate were grown for 6-days in complete RPMI and counted viable cells (million/mL) using Trypan Blue exclusion method in an automated cell counter. **d**, **j** The bar diagrams represent % of apoptosis as determined from subG0 value using propidium iodide (PI) staining of ethanol fixed cells. **c**, **d** and **i**, **j** Error bars represent standard deviations of duplicate assays of two independent experiments. ***p < 0.05

We asked whether EBNA3C mediated cell-cycle activation and apoptotic inhibition can be directly related to autophagy induction. To this end, LCLs knockdown for EBNA3C^38^ and BJAB cells stably expressing EBNA3C (Fig. 1) were subjected to three different analyses—(i) western blots (WB) for cell-cycle (cyclin D1 and pRb), apoptosis (PARP cleavage) and autophagy (LC3II conversion and p62) markers; (ii) cell-proliferation assays, and (iii) cell-cycle analyses to determine subG0 fraction of resting/non-proliferating cells (Fig. 1 and S1). As expected, depletion of EBNA3C expression in LCLs led to a significant reduction in proliferation rate (~3-fold) and increase in subG0 fraction (~15%), accompanied with down-regulation of cyclin D1, up-regulation of pRb and more PARP cleavage (Fig. 1b-e and S1A-C). Conversely, these phenomena were completely reversed in EBNA3C expressing BJAB cells demonstrating acceleration of cell-proliferation rate (~4-fold) and reduction of subG0 fraction (~2.5-fold) when compared to the control cells (Fig. 1i, j, respectively). EBNA3C induced cell-proliferation was similarly validated by elevated expression of cyclin D1, down-regulation of pRb and less PARP cleavage in EBNA3C expressing B-cells (Fig. 1h, l, respectively). Next, increased cell-proliferation and decreased apoptosis (subG0) by EBNA3C were further correlated with autophagy activation markers (Fig. 1f, l). While EBNA3C depletion in LCLs led to decrease in LC3II conversion and increase in p62 accumulation, an opposite phenomenon was observed in EBNA3C expressing B-cells as compared to the control lines (Fig. 1f, l). The results therefore point to a model where EBNA3C induced cell-proliferation and apoptotic inhibition can be directly linked to autophagy activation. Since EBNA3C can transactivate LMP1 expression^25^, it is possible that inhibition of autophagy in EBNA3C knockdown LCLs was due to down-regulation of LMP1 expression. However, as similar to previously published results^39^, our data also demonstrated that EBNA3C knockdown did not affect LMP1 expression (Fig. S2), signifying changes in proliferation, apoptosis, and autophagy were exclusively due to differential expression of EBNA3C.

### EBNA3C protects cell-death induced by autophagy inhibitors

**Fig. 2:**
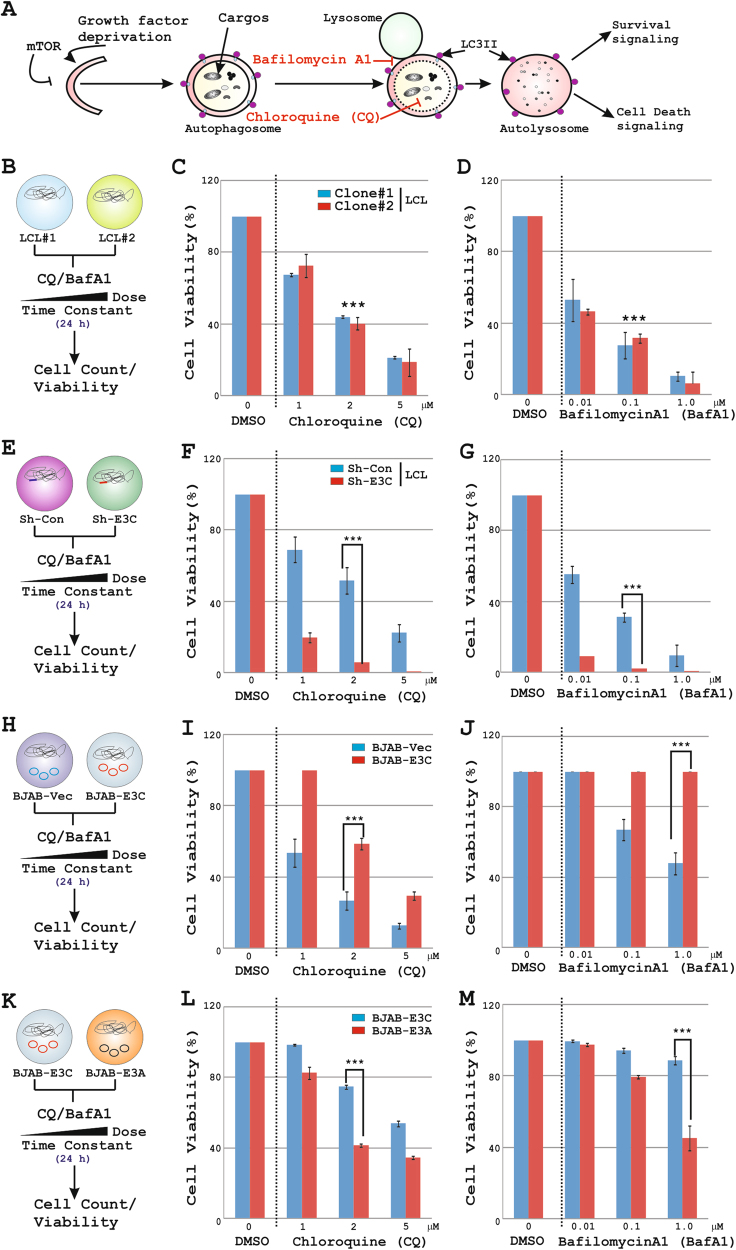
EBNA3C rescues cell-death induced by autophagy inhibitors. **a** Chloroquine (CQ) and Bafilomycin A1 (BafA1) block autophagy flux. ~0.5 × 10^5^ (**b**-**d**) two LCL clones – LCL#1 and LCL#2, **e**–**g** LCLs expressing either sh-control or sh-EBNA3C, **h**–**j** BJAB-vector and BJAB stably expressing EBNA3C, **k**–**m** BJAB stably expressing either EBNA3C or EBNA3A were treated with increasing doses of two different autophagy inhibitory drugs—**c**, **f**, **i**, **l** 0–5 µM CQ and **d**, **g**, **j**, **m** 0 – 1 µM BafA1. 24 h post-incubation cells were subjected to cell viability assay (%; normalized to initial number of untreated cells, DMSO control) using Trypan blue exclusion method in an automated cell counter as described in Fig. 1. Error bars represent standard deviations of duplicate assays of two independent experiments. ***p < 0.05

In many cancers, autophagy appears to play a cytoprotective role that allows uninterrupted cell proliferation under stressful conditions such as starvation or ER-stress due to the unfolded protein response (UPR)^4,40^. To explore whether EBNA3C promotes a cytoprotective autophagy, cells were exposed to increasing concentrations of autophagy inhibitors—chloroquine (CQ) and Bafilomycin A1 (BafA1) and checked for cell viability (Fig. 2). While BafA1, a vacuolar ATPase inhibitor, blocks the autophagosome-lysosome fusion, CQ inhibits autophagy through increasing the lysosomal pH (Fig. 2a)^41,42^. Both CQ and BafA1 treatment caused a drastic reduction in cell viability of two in vitro EBV transformed LCLs (LCL#1 and LCL#2) in a dose dependent manner (Fig. 2b-d), supporting an important role for autophagy activation to maintain LCLs outgrowth. While, EBNA3C knockdown LCLs were more susceptible to cell-death (~3–4 folds) induced by autophagy inhibitors (Fig. 2e-g), EBNA3C expression in B-cells led to a significant protection (~2 folds) in a dose dependent fashion as compared to the control lines (Fig. 2h-j). Since a similar trend of autophagy activation and cytoprotective pattern was repeatedly demonstrated in two EBNA3C expressing clones (#7 and #10) in BJAB cells, we utilized a single EBNA3C expressing line (BJAB-E3C#10) for further experiments (Fig. S3A-E). Although EBNA3A and EBNA3C demonstrate multiple redundant cellular functions^7^, unlike EBNA3C, EBNA3A failed to protect cell-death induced by autophagy inhibitors (Fig. 2k-m). The viral gene expression (EBNA3A and EBNA3C) levels in these stable lines were tested using qPCR and WB analyses (Fig. S4A-C). The primer sequences used for qPCR experiments are described in Table S1. Overall, our data suggests that EBNA3C promoted a cytoprotective autophagy mechanism allowing an uninterrupted cell proliferation (Fig. 2k-m and S4C-D).

### *ATG5* knockdown accelerates cell-death of EBNA3C positive B-lymphocytes in response to autophagy inhibition

**Fig. 3:**
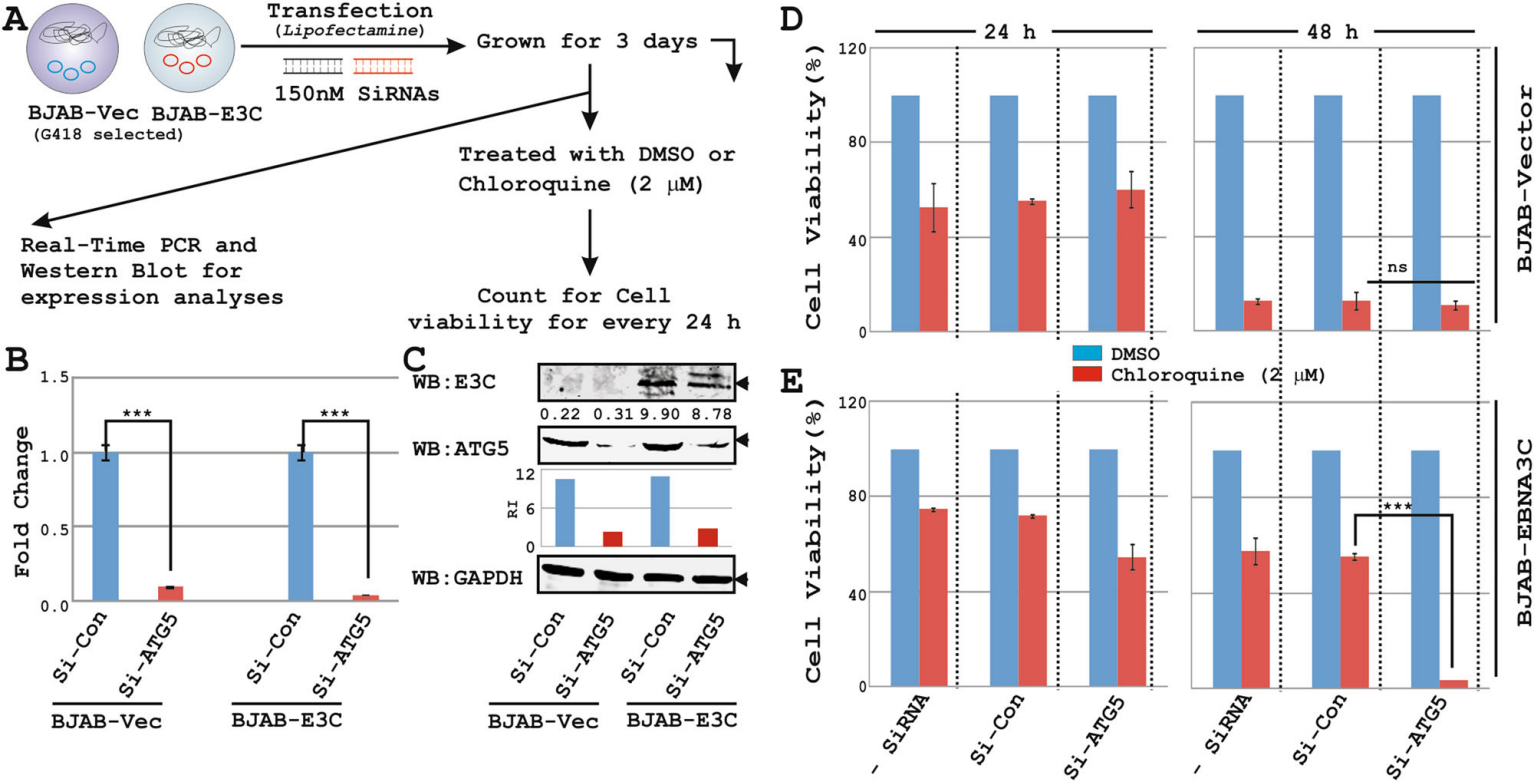
ATG5 knockdown promotes cell-death of EBNA3C expressing B-cells. **a**–**c** ∼1 × 10^6^ BJAB and BJAB stably expressing EBNA3C cells (clone #10) were transfected using specific si-RNAs directed against either *ATG5* or control si-RNAs. Cells were harvested 72 h post-transfection and subjected for (**b**) real-time PCR and **c** WB analyses to check the knockdown efficiency. The relative changes in transcripts using the 2^−ΔΔCt^ method were represented as bar diagram in comparison to control transfected sample using *GAPDH* as a housekeeping gene. Two independent experiments were carried out in similar settings and results represent as an average value with SD. **d** ~0.5 × 10^5^ non-transfected or 72 h post-transfected cells with specific si-RNAs were either left untreated or incubated with DMSO or 2 μM Chloroquine (CQ) for 2 days. After every 24 h viable cells were counted using Trypan Blue exclusion method in an automated cell counter. Average of two independent experiments is represented as bar diagram. ***p < 0.05. ns non-significant

To further elucidate EBNA3C mediated autophagy regulation in maintaining B-cell survival and proliferation, *ATG5* gene was knockdown using naked siRNAs in control and BJAB-EBNA3C cells (Fig. 3a-c) and subjected for cell viability assays in the presence of autophagy inhibitor (CQ) (Fig. 3b, c). Intriguingly, the effect of si-RNA mediated knockdown on cell-death was particularly prominent in EBNA3C expressing B-cells as compared to the control cells (Fig. 3d, e). Typically, EBNA3C expressing cells were more resistant (~2–4 folds) to cell-death caused by the treatment with CQ for 24–48 h in both non-transfected and control si-RNA transfected cells (Fig. 3d, e). The depletion of *ATG5* expression in EBNA3C expressing cells led to increased cell-death when autophagy is inhibited by CQ, while no further cell-death was observed in control knockdown cells (Fig. 3d, e). In contrast, ATG5 knockdown had no effect on EBNA3C mediated protection of cell-death induced by an unrelated drug, thapsigargin (Fig. S5), which causes apoptotic cell-death though inducing UPR^43^. Together, the results indicate that EBNA3C utilizes autophagy pathway to promote B-cell survival.

### EBNA3C elevates autophagy flux

**Fig. 4:**
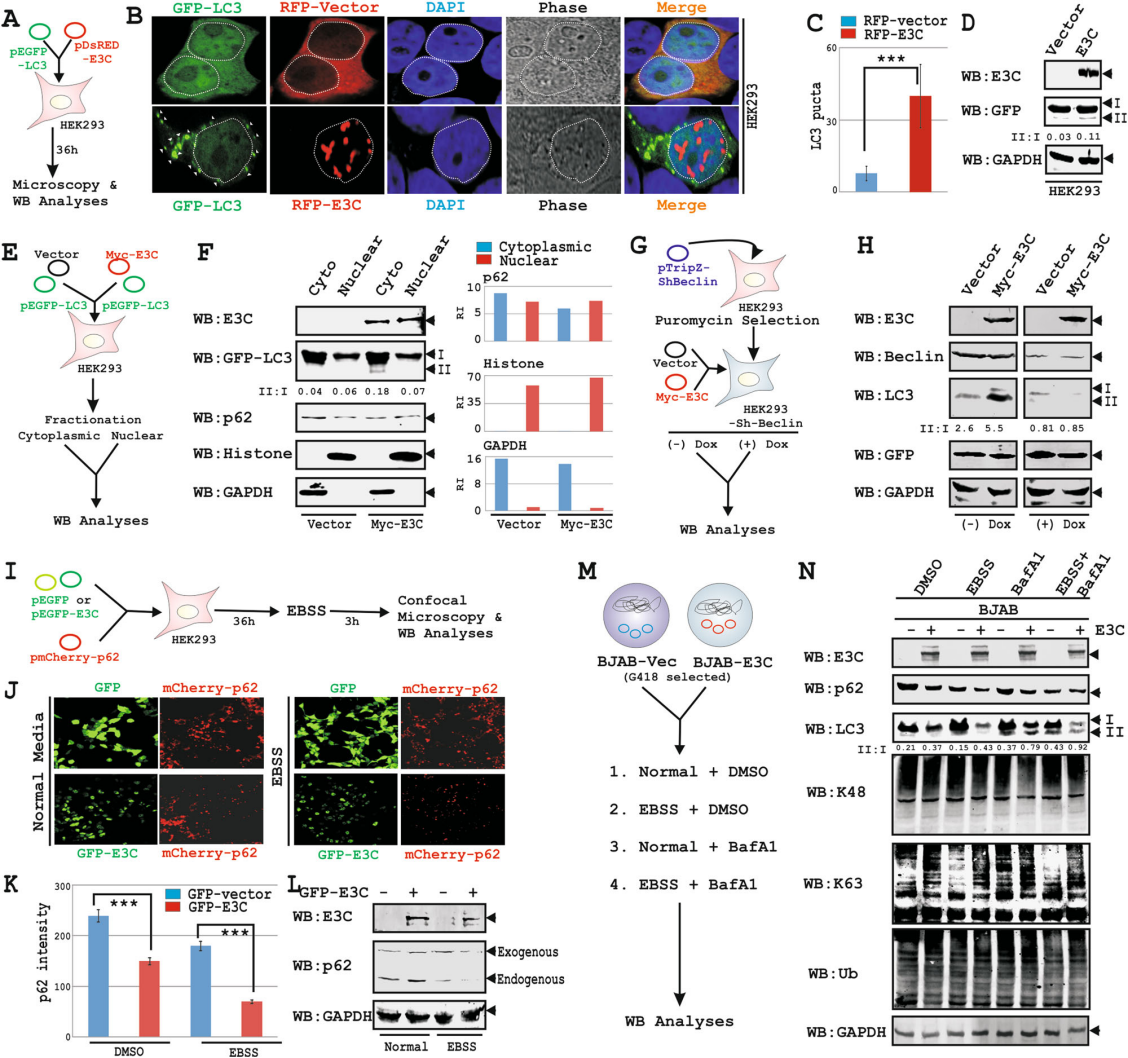
EBNA3C enhances autophagy flux. **a**–**d** ~1 × 10^6^ HEK293 cells co-transfected using Lipofectamine 3000 with indicated plasmid constructs were subjected for either (**b**, **c**) confocal analyses or **d** checking protein expressions. **e**, **f** ~10 × 10^6^ HEK293 cells co-transfected using electroporation with indicated plasmid constructs were harvested after 36 h and subjected for subcellular fractionation as per Manufacturer’s instructions. Fractionated protein samples were analyzed by WB using indicated antibodies. **g**, **h** ~10 × 10^6^ HEK293 cells were transfected with pTripz-Sh-Beclin construct expressing Sh-Beclin under doxycycline (Dox) inducible promoter. Thirty-six hours post-tranfection cells were selected with Puromycin for 4 days. Selected cells were further co-transfected with either pA3M control or myc-EBAN3C expressing plasmids, incubated for 36 h with or without doxycycline and subjected for WB analyses for the indicated antibodies. **i**–**l** ~1 × 10^6^ HEK293 cells co-transfected using Lipofectamine 3000 with indicated plasmid constructs were subjected for either (**b**, **c**) confocal analyses or **d** checking protein expressions using the indicated antibodies. Thirty-six hours post-transfection, cells were either left untreated (DMSO control) or treated with EBSS for another 3 h. **m**, **n** ~10 million BJAB and BJAB stably expressing EBNA3C cells (clone #10) either left untreated (normal growth media) or treated with DMSO, EBSS or bafilomycin (BafA1) separately or in combination (EBSS + BafA1) for 3 h and subjected for WB analyses for the indicated antibodies. **b**, **c** and **j**, **k** For confocal assays ~3–5 × 10^4^ transfected cells were re-plated into each well of a six-well plate containing poly-lysine coated cover-slip, fixed with 1:1 mixture of acetone and methanol. The nuclei were counterstained using DAPI (4’,6’-diamidino-2-phenylindole) for 10 min at room temperature before mounting the cells. The images were sequentially captured using an Olympus confocal microscope. All panels in (**b**, **j**) are representative pictures and in (**c**, **k**) the bar diagram represents the mean value of (**c**) GFP-LC3 puncta from ~50 cells of five different fields of two independent experiments (**k**) p62 puncta intensity, respectively. ***p < 0.05. **d**, **f**, **h**, **l**, **n** GAPDH blots were performed as loading control. Protein band intensities (pixels) were quantified by Odyssey imager software and indicated either as bar diagrams or LC3-II/I ratio at the bottom of each corresponding lane

To verify whether EBNA3C specifically deregulates autophagy, HEK293 cells were transiently transfected with GFP-LC3 plus empty-vector or RFP-EBNA3C expressing plasmids (Fig. 4a-d). In EBNA3C expressing cells the basal level of autophagy was significantly elevated under normal conditions as detected by increasing cytoplasmic puncta of GFP-LC3 in confocal microscopy (Fig. 4b, c), and/or LC3 cleavage pattern in WB analyses (Fig. 4d). A similar phenomenon was also observed under growth limiting conditions (EBSS) (Fig. S6). To corroborate GFP-LC3 distribution in respect to EBNA3C expression, transiently transfected HEK293 cells were fractionated (Fig. 4e, f). The WB results clearly demonstrated an increased LC3 cleavage and reduced p62 accumulation in cytoplasmic fraction of EBNA3C expressing cells (Fig. 4f). The efficiency of subcellular fractionation was checked by GAPDH and Histone blots as cytoplasmic and nuclear reference proteins, respectively (Fig. 4f). To validate EBNA3C mediated basal autophagy induction, HEK293 cells stably knockdown for Beclin1 (expressing sh-Beclin1 under doxycycline inducible promoter), were transiently transfected with empty-vector or myc-EBAN3C expressing constructs (Fig. 4g, h). While in the absence of doxycycline, EBNA3C induced autophagy as indicated by LC3II accumulation, Beclin1 depletion (doxycycline treatment) resulted in inhibition of autophagy pathway irrespective of EBNA3C expression (Fig. 4h).

HEK293 cells transiently expressing mCherry-p62 with or without GFP-EBNA3C were left untreated or incubated with EBSS (Fig. 4i-l) and subjected to confocal microscopy for mCherry-p62 fluorescence intensity (Fig. 4j, k) and WB analyses for p62 degradation (Fig. 4l). The results provided additional support of EBNA3C mediated autophagy induction as detected by enhanced p62 degradation specifically under growth limiting conditions (Fig. 2j-l). To analyze EBNA3C induced autophagy in a more endogenous setting, control and BJAB-EBNA3C cells were further treated with BafA1 with or without growth promoting factors (Fig. 4m, n). EBNA3C mediated induction of the basal level of autophagy was further increased upon incubation with EBSS, and blocked in the presence of BafA1 as indicated by the differential expression pattern of p62 and LC3II (Fig. 4n).

Autophagy inhibition compromises the ubiquitin-proteasome mediated protein degradation^44^. Although both K48-linked and K63-linked poly-ubiquitinated proteins were processed in autophagy when the proteasome was found to be overburden, K63-linked poly-ubiquitinated chains preferentially target substrates for degradation via autophagy^45^. EBNA3C was previously shown to critically modify the ubiquitin-proteasome system^11,28,38^. Interestingly, EBNA3C expression specifically enhanced K63-mediated polyubquitination (Fig. 4n), which was further elevated when autophagy was induced and blocked in the presence of BafA1 (Fig. 4n). However, no significant changes were observed for K48-mediated poly-ubiquitination signal (Fig. 4n). It became apparent that EBNA3C elevates autophagy flux, in which K63-linked polyubiquitinated cargo was captured by the p62 adapter protein and subsequently recycled.

### EBNA3C transcriptionally deregulates the autophagy machinery

**Fig. 5:**
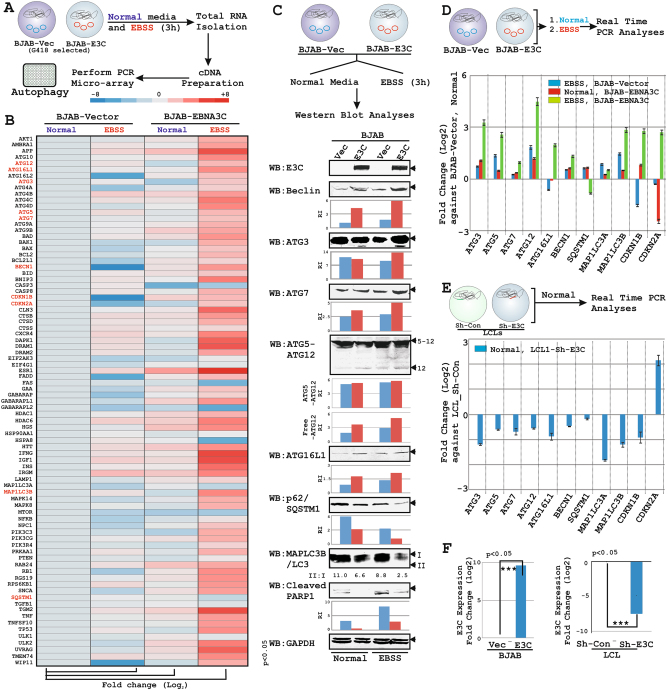
EBNA3C alters autophagy gene transcription under growth limiting conditions. **a**, **b** ∼20 × 10^6^ BJAB-vector and BJAB stably expressing EBNA3C cells (clone #10) either left untreated (normal growth media) or treated with EBSS for 3 h, were harvested for total RNA extraction followed by cDNA generation according to manufacturer’s protocol. Amplified cDNAs were then subjected to (**b**) PCR-microarray analyses for “Autophagy” pathway from SABiosciences in accordance with manufacturer’s instructions. The relative changes in transcripts (log_2_) using the 2^−ΔΔCt^ method were represented as a heat map in comparison to control cells (BJAB-vector, untreated) taking average of five housekeeping genes *ACTB*, *B2M*, *GAPDH*, *HPRT1,* and *RPLP0*. Data analysis (algorithm provided by the manufacturer) also incorporated the quality check to include or exclude the real-time PCR data points for individual genes. Two independent experiments were carried out in similar settings and results represent as an average value for each transcript. Red marked genes were selected for further validation. **c**, **d** ∼10 × 10^6^ BJAB control and BJAB stably expressing EBNA3C cells (clone #10) either left untreated (normal growth media) or treated with EBSS for 3 h, were harvested and subjected for either (**c**) WB analyses or (**d**) real-time PCR analyses for the selected gene expressions. **c** Protein band intensities (pixels) in each lane were quantified by Odyssey imager software and presented as either by bar diagrams at the bottom of each picture. **e** ∼10 × 10^6^ control and EBNA3C knockdown LCLs were harvested and subjected for real-time PCR analyses for the selected genes. The relative changes in transcripts (log2) using the 2^−ΔΔCt^ method were represented as bar diagram (log_2_ scale) in comparison to either (**d**) BJAB control or (**e**) LCL-Sh-control samples. **f** EBNA3C transcripts were measured and plotted as bar diagrams in a similar experimental set-up described in both **d** and **e**. In all real-time PCR experiments *GAPDH* was used as a housekeeping gene. Two independent experiments were carried out in similar settings and results represent as an average value for each transcript

EBNA3C is a non-DNA binding transcriptional regulator that modulates a number of viral and cellular gene expressions^7,8^. To explore the overall transcriptional changes in autophagy and related pathway in response to EBNA3C expression, we utilized PCR-microarray platform profiling a total of 84 key autophagy regulatory genes (Table S2). cDNA generated from control and BJAB-EBNA3C cells with or without EBSS treatment were subjected to PCR microarray analyses (Fig. 5a, b). The relative expression (ΔCt value) of each transcript from average value of five housekeeping genes in the array platform was represented as heatmap using 2^−ΔΔCt^ method after normalization of the raw data in respect to control treated cells (Fig. 5b). Under growth limiting conditions, EBNA3C expression drastically enhanced transcription of several autophagy and other related signaling molecules engaged in cell-cycle and apoptotic pathways (Fig. 5b). Notably, a number of death promoting tumor suppressor genes (viz. *CDKN2A*, *CDKN1B*, *DAPK1*) were also markedly elevated in EBNA3C expressing cells under growth limiting conditions (Fig. 5b).

To validate the PCR microarray data, WB analyses was performed of eight deregulated autophagy markers including ATG3, ATG5, ATG7, ATG12, ATG16L1, Beclin1 (*BECN1*), p62 (*SQSTM1*), and LC3 (*MAP1LC3B*) using a similar experimental set up (Fig. 5c). EBSS treatment led to increased autophagy in EBNA3C expressing B-cells as indicated by depletion of p62 and LC3I levels (Fig. 5c). Importantly, the essential components of autophagosome formation such as ATG3, ATG5-ATG12 complex, ATG7, ATG16L1, and Beclin1 were also elevated in EBNA3C expressing cells upon autophagic induction (Fig. 5c). We also performed real-time PCR experiments of similar set of genes along with two other tumor suppressor genes *CDKN2A* (p16^INK4a^) and *CDKN1B* (p27^Kip1^) under similar conditions (Fig. 5d). As similar to PCR microarray, qPCR data also demonstrated that EBNA3C specifically enhanced transcription of *ATG3*, *ATG5*, *ATG7*, *ATG12*, *ATG16L1*, *MAP1LC3A*, *MAP1LC3B*, *CDKN2A*, and *CDKN1B* specifically under growth limiting conditions (Fig. 5d).

EBNA3C mediated transcriptional regulation of ATGs were also evaluated in control and EBNA3C knockdown LCLs (Fig. 5e). Overall, the results demonstrated EBNA3C mediated transcriptional deregulation of autophagy genes along with two CDK inhibitors p16^INK4A^ and p27^KIP1^ (Fig. 5b, d, e). While as expected from previously published data^14^, EBNA3C expression led to a transcriptional deactivation of *CDKN2A* gene, an unusual but significant positive correlation with *CDKN1B* transcript was determined under normal conditions (Fig. 5d, e). However, *CDKN2A* and *CDKN1B* transcripts were significantly upregulated in EBNA3C expressing B-cells under serum/amino acids starved conditions (Fig. 5b, d). EBNA3C transcript was analyzed by qPCR in these cell lines (Fig. 5f). Taken together, the data suggest that EBNA3C expression leads to a modest increase in autophagy and partial decrease in apoptosis related gene expressions in the presence of growth promoting signals, while during metabolic stress conditions both autophagy and apoptotic gene expressions are elevated.

### EBNA3C recruits histone activation epigenetic marks for autophagy gene transcription

To determine the underlying mechanism through which EBNA3C regulates autophagy gene transcription, we reanalyzed the previous EBNA3C ChIP-seq data (GEO dataset ID: GSE52632^14^) for all the 84 genes from PCR-microarray platform (Table S3). Additionally, to identify EBNA3C mediated epigenetic landscape among deregulated autophagy genes, EBNA3C peaks were further aligned with the data from ENCODE (Encyclopedia of DNA Elements) GM12878 LCL (GEO dataset ID: GSE26386^36^) for five different histone modifications—H3K4me1, H3K4me3, H3K9ac, H3K27ac, and H3K27me3 (Tables S4-S7. However, no clear H3K27me3 peaks were identified for the selected gene loci). Among these histone modification signals, H3K4me1, H3K4me3, H3K9ac, and H3K27ac symbolize active transcription^36^. While H3K4me1 is primarily associated with enhancers, H3K4me3 engages at promoter sites and both H3K9ac and H3K27ac represent an active regulatory region of the gene^36^. Conversely, H3K27me3 denotes transcriptional repression associated with polycomb repressive complex (PRC)^36^. Earlier studies revealed that epigenetic modifications regulate the autophagic process^46^. EBNA3C was also shown to regulate gene transcription connecting through epigenetic machineries—ranging from recruiting several HATs and HDACs to various epigenetic histone modifications^14,21,26,47^. It is therefore tempting to speculate that EBNA3C may also engage epigenetic modifications to control autophagy gene transcription.

**Fig. 6:**
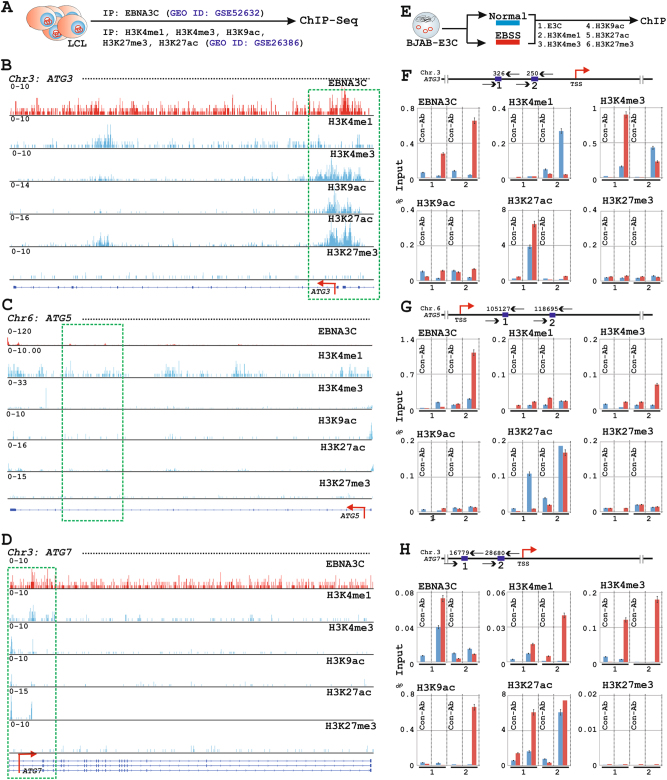
EBNA3C recruits histone activation epigenetic marks to regulate *ATG3*, *ATG5*, and *ATG7* gene loci. **a**–**d** ChIP-seq data (GEO dataset IDs: GSE52632 for EBNA3C and GSE26386 for histone epigenetic modifications—H3K4me1, H3K4me3, H3K9ac, H3K27ac, and H3K27me3) were reanalyzed and displayed using IGV (Integrative Genomics Viewer) software for (**b**) *ATG3*, **c** *ATG5*, **d** *ATG7* gene loci. **e**–**h** Chromatin immunoprecipitation (ChIP) was performed using ~5 × 10^6^ BJAB stably expressing EBNA3C cells (Clone #10) after treatment with either DMSO (control) or EBSS for 6 h using indicated antibodies on (**f**) *ATG3*, **g** *ATG5*, **h** *ATG7* gene loci. Binding sequences are taken from the ChIP-seq data for individual gene locus and ChIP primers are designed by NCBI primer BLAST application. For ChIP-PCR, two independent experiments were carried out in similar settings and results represent as an average value for each genomic segment. Transcription start sites (TSS) are indicated by red arrows

**Fig. 7:**
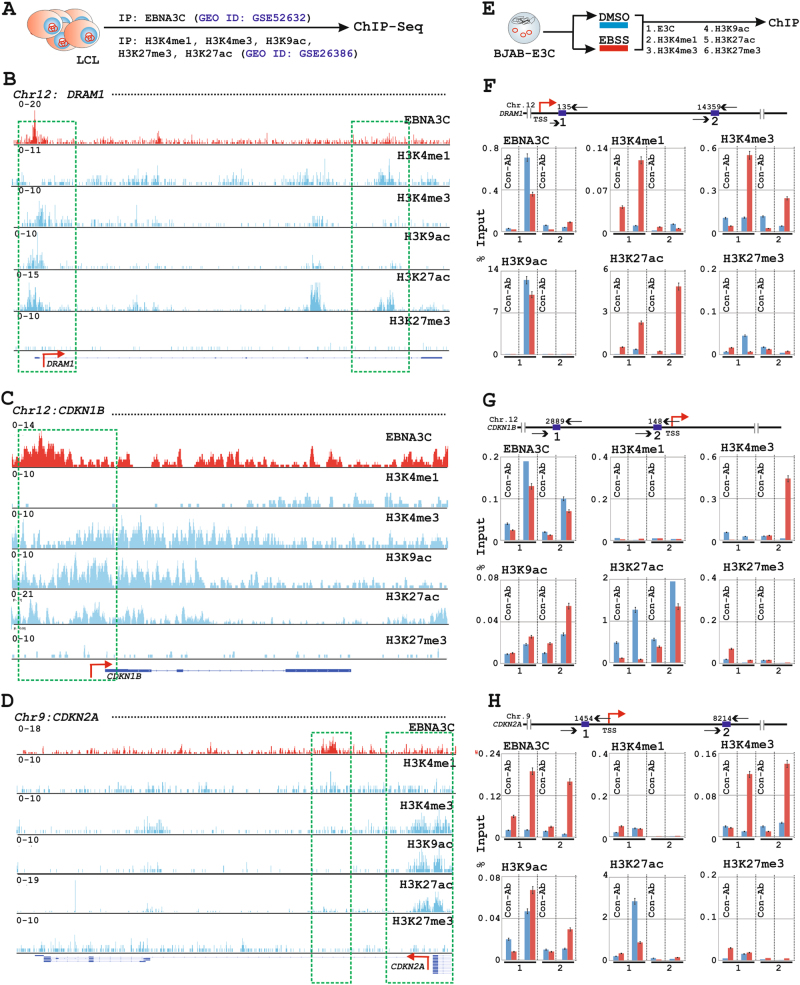
Distribution of EBNA3C binding and different histone modification sites around *DRAM1*, CDKN1B, and *CDKN2A* gene loci. **a**–**d** As described in Fig. 6, ChIP-seq data were reanalyzed and displayed using IGV software similarly for (**b**) *DRAM1*, **c** *CDKN1B*, **d** *CDKN2A* gene loci. **e**–**h** ChIP-PCR was performed using parallel sample as described in Fig. 6 on (**f**) *DRAM1*, **g** *CDKN1B*, **h** *CDKN2A* gene loci. Binding sequences are taken from the ChIP-seq data for individual gene locus and ChIP primers are designed by NCBI primer BLAST application. For ChIP-PCR, two independent experiments were carried out in similar settings and results represent as an average value for each genomic segment. Transcription start sites (TSS) are indicated by red arrows

Reanalysis of EBNA3C ChIP-Seq data revealed that EBNA3C peaks were significantly enriched on a number of ATGs (Fig. 5 and Table S3 for ChIP-Seq calculations). These genes include *ATG3*, *ATG5*, *ATG7*, *ATG10*, *UVRAG*, *PIK3C3,* and *PIK3CG* involved in autophagosome biogenesis; CDKis *CDKN1B* and *CDKN2A*; tumor suppressor protein *RB1*; DNA damage induced autophagy mediated death regulators *DRAM1 and DAPK1* (Figs. 6 and 7a–d, S7-S8A-D, red histograms and Table S3). Interestingly, most of these identified EBNA3C peaks were found to be intragenic near the enhancer regions and very few were located at upstream promoter region (Table S3). For example, strong EBNA3C signals were observed at the promoter regions of both CDKis *CDKN1B*, *CDKN2A,* and *DRAM1* (Fig. 7). To assess EBNA3C activated or repressed transcription, histone epigenetic on EBNA3C bound ChIP-Seq signals were further analysed with ENCODE GM12878 LCL histone ChIP-Seq data (Figs. 6 and 7a–d, S7-S8A-D, blue histograms). In agreement with the transcriptomics data, the ChIP-Seq analyses revealed that EBNA3C engaged itself in active transcription of the autophagy machinery as characterized by H3K4me1, H3K4me3, H3K9ac, and H3K27ac marks. For instance, EBNA3C bound multiple downstream regions at *ATG5* loci (+103 bp, and +52 to +141 kb) coincided with H3K4me1 signals indicative of active enhancers, and upstream region at *ATG3* loci (−326 bp) overlapped with H3K4me3, H3K9ac, and H3K27ac marks characteristic of active promoter (Fig. 6b, c). Moreover, to validate the ChIP-Seq data and to rule out the involvement of other viral latent oncoproteins, ChIP assay was performed using BJAB-EBNA3C cells with or without EBSS (Figs. 6 and 7e–h). ChIP primers were designed as indicated in the cartoon diagrams based on two consistent EBNA3C binding sites on both ChIP-Seq replicates (Table S8). Overall, the results indicated that during metabolic stress condition, EBNA3C recruited histone chromatin activation marks not only to enhance transcription for ATGs such as *ATG3*, *ATG5*, *ATG7* but also accelerate various other stress induced cell-death regulators like *DRAM1*, *CDKN2A*, *CDKN1B* (Figs. 6 and 7e–h).

**Fig. 8:**
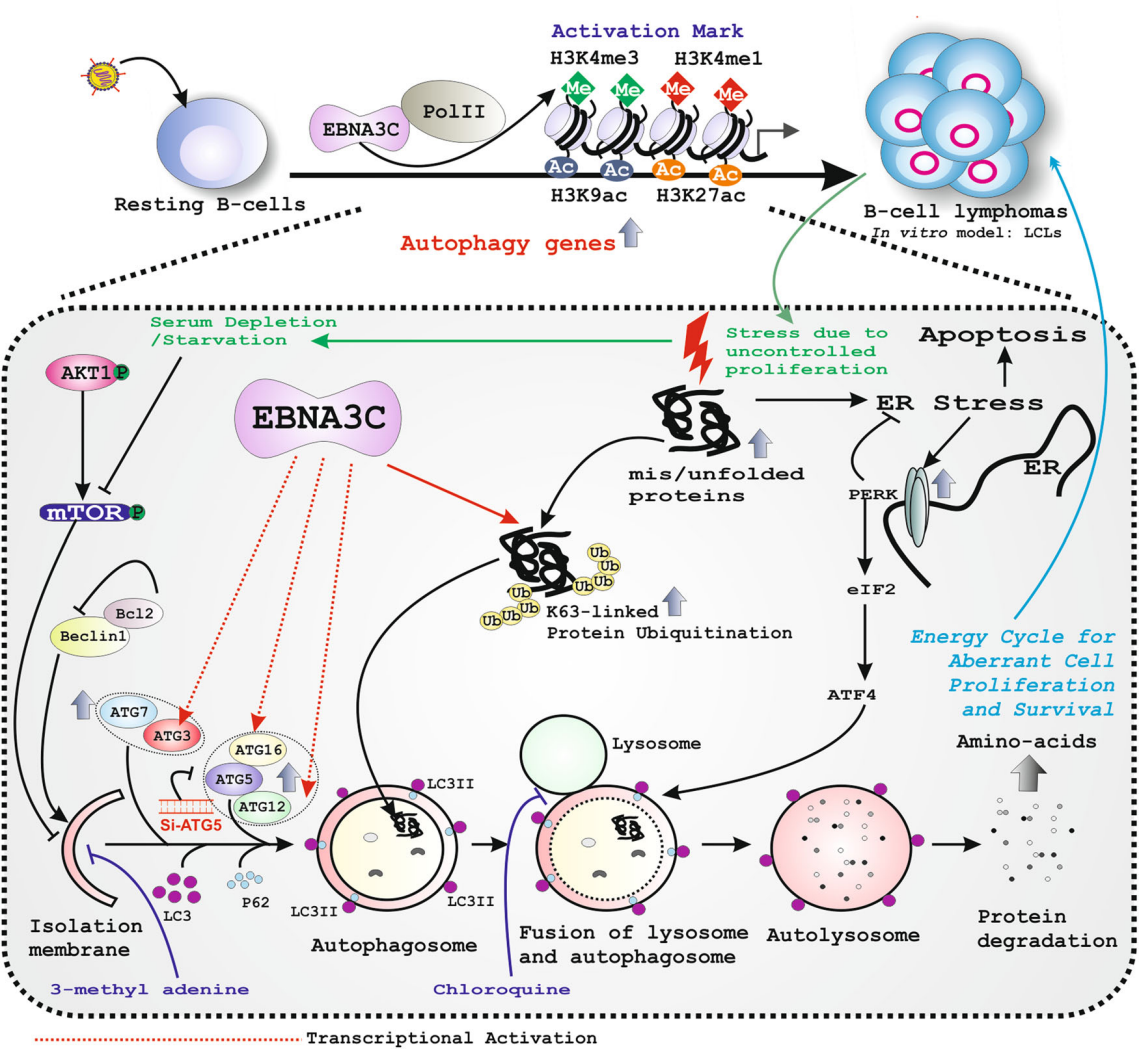
Model depicts EBNA3C deregulates autophagy-UPR network for B-cell survival. Schematic representation of EBNA3C induced autophagy. Upon EBV infection in resting B-cells EBNA3C recruits histone activation chromatin marks (H3K4me1, H3K4me3, H3K9ac, and H3K27ac) to elevate a number of autophagy related gene transcriptions such as *ATG3*, *ATG5*, *ATG7,* and *ATG16* under growth limiting conditions. ATG3, ATG5, ATG7, and ATG16 assist in autophagosome formation through inducing LC3-lipidation. p62 acts as an adaptor molecule collecting poly-ubiquinated cargo meant to be degraded in lysosomal compartments. Bcl2 is an anti-apoptotic protein, which blocks Beclin-1 activity through direct protein-protein interaction, providing a feedback regulation. EBNA3C mediated autophagy activation provides an alternative energy source through degradation of K63-linked polyubiquitinated protein aggregates for aberrant cell proliferation and subsequent B-cell lymphoma development

All together, the results suggest a model in which EBNA3C enhances autophagy by transcriptional upregulation of a number of autophagy genes specifically required for autophagosome biogenesis to cope with several cellular insults such as depletion of growth factor signaling due to aberrant cell proliferation of EBV transformed B-lymphocytes (Fig. 8).

## Discussion

A growing body of evidence has shown that several viral antigens deregulate autophagy either by directly interacting with autophagy-related proteins or by modulating the diverse signaling cascades that affect autophagy^29^. In this study we demonstrated that EBV oncoprotein EBNA3C upregulates basal autophagy, which is further elevated in growth limiting conditions as determined by elevation of several autophagy markers such as increased LC3-II conversion, upregulation of ATG3, ATG5, and ATG7 molecules directed for autophagosome formation and accumulation of K63-linked ubiquitinated protein aggregates along with p62 degrdation. Autophagy plays an important role for presenting antigenic epitopes on MHC class II for CD4+ T-cell recognition^29,48,49^. It has been shown that while EBNA1 was processed through autophagy, fascinatingly two other EBV antigens EBNA2 and EBNA3C circumvent the process without any apparent explanation^34,35^. It is still not clear how EBNA3C induced autophagy offers an immune escape strategy in MHC class-II positive B-lymphocytes. Importantly, many herpesviruses evolve multiple strategies to evade autophagy-mediated host antiviral defense mechanism and thereby promoting virus-induced oncogenesis^30^. For example, both KSHV and HVS (herpesvirus saimiri) encoded vFLIP, a viral homolog of cFLIP, suppresses autophagosome formation through direct interaction with the E2-like ATG3^50^. Similarly, KSHV and MHV-68 encoded vBCL2 inhibits autophagy in a very similar way to its cellular counterpart BCL2 through targeting Beclin1 during initiation of the autophagosome formation^51^. Unlike these herpesviral proteins, EBV oncoprotein LMP1 activates autophagy that in turn regulates B-cell physiology^33,52^.

In response to various stresses such as nutrient and amino-acids depletion or hypoxia autophagy is activated independent or dependent of transcriptional regulation. In fact, the cytoplasmic mechanism governing autophagy has been extensively studied^53^, whereas nuclear regulation remains largely undefined. Based on the localized histone modification patterns, chromatin is organized into either permissive or repressive mode for gene transcription via recruiting various transcription factors^54^. Recent studies unveiled several stress-responsive transcription factors including ATF4, E2F1, p53, p73, CHOP, C/EBPβ, FOXO3, STAT3, and NF-κB that regulate autophagy^53^. Moreover, histone modifications particularly H4K16ac and H3K9me2 and microRNAs can also affect autophagy gene expressions^46^. Our results indicate that EBNA3C engages multiple histone activation marks—H3K4me1, H3K4me3, H3K9ac, and H3K27ac to regulate autophagy gene transcriptions. In addition to these histone modifications, finding other epigenetic modifiers and novel transcriptional regulators, will provide further insights into how EBNA3C modulates the autophagy gene transcription program in EBV infected B-lymphocytes.

In agreement with the previous findings, our results also signify an intricate connection between apoptosis and autophagy cascades. For example, p53 plays an important role in autophagy regulation^53,55^. Although in response to various genotoxic stresses, nuclear localized p53 promotes autophagy through transcriptional activation of many ATGs, typically p53 inhibits autophagy in cancer cells^56^. In fact, depletion of p53 expression enhances autophagy^53^. Several viral oncoproteins including EBNA3C block p53 induced apoptosis^8,27^. However, whether this contributes to autophagy deregulation remains elusive. Our results demonstrate that under nutrient deprivation, EBNA3C transactivates p53 regulated autophagy gene *DRAM1*, implying a dual role for EBNA3C in regulating apoptosis and autophagy depending on the extracellular stimuli. Unveiling the molecular mechanism by which DRAM1 regulate autophagy would certainly provide a better understanding of EBNA3C-modulated p53 functions in EBV-associated B-cell lymphomagenesis leading to targeted therapy.

CDKN1B (p27^Kip1^) blocks cell proliferation and induces apoptosis in the absence of growth promoting signals. In many cancers p27^Kip1^ expression was shown to be significantly downregulated^57^. Conversely, p27^Kip1^ can also induce autophagy in cancer cells^58^. Under metabolic stress condition, the nutrient-sensing LKB1-AMPK pathway stabilizes p27^Kip1^ at cytoplasm^59^. However, it remains elusive whether p27^Kip1^ directly regulates autophagy machinery or via recruiting other autophagy regulators. p27^Kip1^ expression is regulated by transcription, translation, phosphorylation, and ubiquitin-mediated proteolysis^57^. EBNA3C facilitates p27^Kip1^ degradation by recruiting SCF^Skp2^ E3-ligase activity^24^. Here, we show that EBNA3C transcriptionally activates p27^Kip1^ expression and that in turn may induce autophagy during metabolic stress conditions. Future studies will be required to address the precise mechanism by which p27^Kip1^ links autophagy activation in EBV infected B-lymphocytes. In agreement with the previous study^14^, our results also demonstrate that EBNA3C transcriptionally deactivate CDKN2A (p16^INK4a^). Importantly, overexpression of several CDKis including p16^INK4a^, induce autophagy and senescence along with reduced tumor growth^60^. The precise molecular mechanism that governs p16^INK4a^ mediated senescence, apoptosis, and autophagy is not fully understood. Importantly, autophagy was shown to block senescence in maintaining the stemness property of human geriatric satellite cells^61^. It is therefore conceivable that EBNA3C induced autophagy may also negatively regulate overall senescence as a countermeasure and thereby energizing cells for incessant proliferation. Nevertheless, our data clearly indicate that EBNA3C plays a multifaceted role in regulating different functions exerted by these CDKis in response to various stresses.

Recently, DAPK1, a member of serine/threonine kinase family, has been implicated in various cellular functions including apoptosis and autophagy through independent mechanisms^62^. Under metabolic stress conditions, DAPK1 promotes autophagy through interacting with MAP1B, a microtubule associated protein, that negatively regulates autophagy^63^. Moreover, DAPK1 expression was recently shown to be linked with one of the key ER-stress regulated transcription factors ATF6^64^. Interestingly, LMP1 elevates DAPK1 expression^65^. However, whether LMP1 mediated DAPK1 regulation leads to autophagy activation is not known. Our data also showed that EBNA3C transcriptionally activates DAPK1 exclusively in the absence of growth promoting signals. Of note, DAPK1 induces cell-death in a p53-dependent pathway where cells contain wild-type p53, while in p53 mutant cells (as in case of BL) it promotes cell growth^66^, suggesting a potential mechanism of EBNA3C mediated autophagy and apoptotic regulation.

Overall, the current study represents an initial step towards understanding yet another critical mechanism employed by the EBV oncoprotein EBNA3C. We certainly expect future therapeutic expansion targeting autophagy network against multiple EBV-associated B-cell lymphomas generated in an immune-compromised scenario.

## Materials and methods

### Ethics statement

Human peripheral blood mononuclear cells (PBMCs) were isolated from unidentified donors, with informed and written consent, which is approved by ‘Institutional Ethics Committee (IEC) for Research on Human Participant’, Presidency University, Kolkata, India based on Helsinki recommendations.

### Cell lines, plasmids, and antibodies

HEK293 cells were obtained from Rupak Dutta (Indian Institute of Science Education and Research, Kolkata, India). HEK293T cells were purchased from GE Healthcare Dharmacon Inc., Lafayette, CO, USA. Both HEK293 and HEK293T cells were maintained in Dulbecco’s modified Eagle’s medium (DMEM) (Gibco/Invitrogen, Inc., USA) supplemented with 10% fetal bovine serum (FBS) (Gibco/Invitrogen, Inc., USA), 1% Penicillin-Streptomycin Solution (Sigma-Aldrich Corp., St. Louis, MO). EBV-negative BL lines BJAB and BJAB stably expressing EBNA3A cells were obtained from Elliott Kieff (Harvard Medical School, USA). In vitro EBV transformed LCL clones (LCL#1 and LCL#2), EBV-negative BL line BJAB, BJAB clones stably expressing either vector or EBNA3C cDNA (clones: E3C#7 and E3C#10), and lentivirus-mediated stable EBNA3C knockdown (LCL#sh-EBNA3C) or control (LCL#sh-ctrl) lymphoblastoid cell lines (LCLs) were previously described^38^. BJAB-vector, BJAB-EBNA3A, and BJAB-EBNA3C cells were maintained in complete RPMI media supplemented with 500 μg/ml G418 (Sigma-Aldrich Corp., St. Louis, MO). LCL#sh-control and LCL#sh-EBNA3C cells were maintained in complete RPMI media supplemented with 1 μg/ml puromycin (Sigma-Aldrich Corp., St. Louis, MO). LCLs were maintained in RPMI 1640 (Gibco/Invitrogen, Inc., USA) supplemented with 10% FBS and 1% Penicillin-Streptomycin Solution. Unless otherwise stated all above-mentioned cells were cultured at 37 °C in a humidified environment supplemented with 5% CO_2_.

Myc-tagged EBNA3C construct in pA3M vector and GFP-tagged EBNA3C in pEGFP vector were previously described^11,24^. pA3M-EBNA3C was used as a template for preparing C-terminal red fluorescence protein (RFP)-tagged EBNA3C expressing construct by cloning PCR-amplified fragment into pDsRED-Monomer-N1 (Clontech Laboratories, Inc.) at HindIII and SalI restriction sites. The construct was subsequently verified by DNA sequencing (Eurofins Genomics India Pvt. Ltd., India). pEGFP-C1 vector was obtained from Clontech Laboratories, Inc. mCherry-tagged p62 expressing plasmid was a kind gift from Edward M. Campbell (Loyola University Chicago, IL, USA). pBabePuro-GFP-LC3 construct was a kind gift from Jayanta Debnath (Addgene plasmid# 22405^67^). pTripz-Sh-Beclin construct (Clone ID: V2THS_23692) was obtained from GE Healthcare Dharmacon Inc., Lafayette, CO, USA.

Mouse monoclonal antibodies reactive to EBNA3C (A10) and myc epitope (9E10) generated from corresponding hybridoma cells were previously described^38,68^. Sheep polyclonal anti-EBNA3C (ab16128), rabbit polyclonal anti-GFP (ab290), rabbit anti-Sheep IgG (H + L), ChIP grade rabbit polyclonal anti-Histone H3 acetyl lysine 27 (H3K27ac; ab4729) antibodies were obtained from Abcam (Cambridge, UK). Mouse monoclonal antibodies directed against p62/SQSTM1 (Clone# 3/P62 lck ligand), Beclin1 (Clone# 20/Beclin), cleaved PARP (Clone# Asp214), Cyclin D1 (Clone# DCS-6), retinoblastoma protein (pRb) (Clone# G3-245), β-actin ab-5 (Clone# C4/*actin*), total ubiquitin (Clone# 6C1.17) were purchased from BD Biosciences (Franklin Lakes, NJ, USA). Rabbit monoclonal antibodies directed against LC3A/B (Clone# D3U4C), ATG5 (Clone# D5F5U), ATG12 (Clone# D88H11), ATG16L1 (Clone# D6D5), ATG7 (Clone# D12B11), and rabbit polyclonal antibody against ATG3, K48-linkage specific polyubiquitin (D9D5) and K63-linkage specific polyubiquitin (D7A11) were purchased from Cell Signaling Technology Inc. (Danvers, MA). Mouse monoclonal anti-GAPDH and anti-cMyc antibodies were purchased from Sigma-Aldrich Corp. (St. Louis, MO, USA). Rabbit polyclonal ChIP grade antibodies against histone H3 acetyl lysine 9 (H3K9ac), histone H3 methyl-lysine 4 (H3k4me1), histone H3 tri-methyl-lysine 4 (H3k4me3) and DyLight 800/680 conjugated secondary antibodies anti-mouse, anti-rabbit, anti-sheep IgG (H + L) were purchased from Thermo Fisher Scientific Inc. (Waltham, MA).

### Western blotting

Ten million transiently transfected adherent or B-cells were harvested, washed with ice cold 1× PBS (Gibco/Invitrogen, Inc., USA), and subsequently lysed in 0.5 ml ice cold RIPA buffer (Sigma-Aldrich Corp., St. Louis, MO) with protease inhibitor (Cell Signaling Technology Inc., Danvers, MA). Protein samples were estimated by Bradford reagent (BIO-RAD, Hercules, CA). Samples were boiled in laemmli buffer (BIO-RAD, Hercules, CA), fractionated by SDS-PAGE and transferred to a 0.45 mm nitrocellulose membrane (BIO-RAD, Hercules, CA). The membranes were then probed with specific antibodies followed by incubation with appropriate infrared-tagged/DyLight secondary antibodies and viewed on an Odyssey CLx Imaging System (LiCor Inc., Lincoln, NE). Image analysis and quantification measurements were performed using the Odyssey Infrared Imaging System application software (LiCor Inc., Lincoln, NE).

### Real-time quantitative PCR (qPCR)

Total RNA was isolated from approximately 10 million cells using TRIzol reagent according to the manufacturer’s instructions (Invitrogen Inc., CA), followed by cDNA preparation using iScript cDNA synthesis kit (BIO-RAD, Hercules, CA) as per manufacturer’s protocol. RNA and cDNA quality and quantity was measured using Synergy™ H1 Multimode Microplate Reader (BioTek Instruments, Inc., VT, USA). qPCR analysis was performed using SsoFast EvaGreen Supermix (BIO-RAD, Hercules, CA) in CFX Connect™ real-time PCR detection system (BIO-RAD, Hercules, CA) with the following thermal profile—1 cycle: 95 °C for 10 min; 40 cycles: 95 °C for 15 s followed by 60 °C for 1 min; and finally the dissociation curve at – 95 °C for 1 min, 55 °C 30 min, and 95 °C for 30 s. Unless and otherwise stated, each sample was performed in duplicate and calculation was made using a 2^−ΔΔCT^ method to quantify relative expression compared with housekeeping gene control. The relative change in the gene expression levels compared to control sample (untreated) was represented as heat map diagram. The primers used in this study were designed from qPrimerDepot (https://primerdepot.nci.nih.gov/) and listed in Tables S1 and S2. Real-time PCR primers were obtained from Integrated DNA Technologies, Inc. (Coralville, IA, USA).

### Transfection

Approximately 10 × 10^6^ HEK293 cells were transfected by electroporation using a Gene Pulser II electroporator (BIO-RAD, Hercules, CA) at 210 V and 975 µF for Western Blot analyses. In case of confocal microscopy and siRNA mediated knockdown analyses cells were transfected using Lipofectamine 3000 and RNAiMAX, respectively according to manufacturer’s protocol (Invitrogen, Thermo Fisher Scientific Inc., Waltham, MA).

### Generation of EBNA3C knockdown LCLs

Control sh-RNA (5′-TCTCGCTTGGGCGAGAGTAAG-3′) and sh-RNA directed against EBNA3C ORF (CCATATACCGCAAGGAAT) were designed (Dharmacon Research, Chicago, IL, USA) and obtained from Integrated DNA technologies, Inc., Iowa, USA. Both the sense and antisense strands of the sh-RNA oligos were annealed and subsequently cloned in pGIPZ vector (Open Biosystems, Inc. Huntsville, AL) using *Xho*I and *Mlu*I restriction sites. Lentivirus production and transduction of EBV-transformed B-cells (LCLs) were essentially carried out as previously described^38^. Briefly, approximately 70% confluent HEK293T cells were transfected with 20 μg of total plasmid DNAs consisting of 1.5 μg pCMV-VSV-G (Addgene plasmid# 8454), 3 μg pRSV-Rev (Addgene plasmid# 12253), 5 μg pMDLg/pRRE (Addgene plasmid# 12251), and 10.5 μg either control sh-RNA or sh-EBNA3C expressing pGIPZ vector. To stabilize the DNA constructs 25 μM chloroquine was used 5 min prior to transfection. Twelve hours post-transfection, the medium was changed with fresh DMEM supplemented with 10 mM sodium butyrate for virus induction for 8 h. Lentivirus was collected twice per day (every 10–12 h) for 2 days in RPMI 1640 supplemented with 10% FBS and 10 mM HEPES, filtered through 0.45 μM filters (Corning Inc., NY, USA) to avoid any cell contamination. The supernatant containing virus was concentrated (100×) by ultra-centrifugation at 70,000 × *g* for 2.5 and subsequently used for infection in the presence of 8 μg/ml Polybrene (Sigma-Aldrich Corp. St. Louis, MO). Three-days post-infection cells were selected using 2 μg/ml puromycin (Sigma-Aldrich Corp. St. Louis, MO) for ~3–4 weeks. Selected cells were grown in complete RPMI supplemented with 1 μg/ml puromycin for at least 1 month before subjected to any further experiments (cell viability and western blot analyses).

### Proliferation assay

0.1 × 10^6^ over 95% viable either BJAB and BJAB stably expressing EBNA3C or LCLs expressing Sh-control or Sh-EBNA3C cells plated into each well of a six-well plate were grown for 6 days in the presence of corresponding antibiotics. Viable cells were counted at 0, 2, 4, and 6 days with Trypan Blue exclusion technique in an automated cell counter as previously described. The experiments were repeated twice and represented as an average value.

### Determination of SubG0 value

Approximately 1 × 10^6^ BJAB and BJAB stably expressing EBNA3C or LCLs stably knockdown for EBNA3C and control cells were harvested, washed with 1× PBS and fixed with ice-cold 70% ethanol (Merck Millipore). Fixed cells were stained with 1× PBS containing 10 μg/mL of propidium iodide (PI), 250 μg/mL of RNase A (Sigma-Aldrich Corp. St. Louis, MO) and 0.05% of Triton X-100 for 1 h at room temperature in dark. Stained cells were analyzed on FACScalibur cytometer and Cellquest software (Becton-Dickinson Inc., San Jose, CA). The subG0 value from two independent experiments were averaged and represented as bar diagram.

### Cell viability assay

Approximately 0.5 × 10^5^ cells plated into each well of the six-well plates (Corning Inc., NY, USA) were treated with different drugs at various concentrations for the indicated time points at 37 °C in a humidified CO_2_ chamber. Autophagy inhibitors including 3-methyl adenine (3-MA) and Chloroquine (CQ) and UPR inducer thapsigargin were purchased from Sigma-Aldrich Corp. St. Louis, MO. Twent-four hours post-treatment, viable cells from each well were measured by Trypan blue exclusion method using an automated cell counter (BIO-RAD, Hercules, CA). Experiments were performed in duplicate and were independently repeated two times.

### Si-RNA mediated knockdown

Small interfering RNAs (si-ATG5 #s18160; si-control #4390843) were obtained from Invitrogen (Thermo Fisher Scientific, Waltham, MA). Si-RNA transfection was conducted according to manufacturer’s protocol with few modifications. Briefly, ~1 × 10^6^ cells seeded in six-well plate in RPMI medium without antibiotics for overnight were transfected with 200 pmol of respective siRNA duplex using Lipofectamine RNAiMAX Reagent (Invitrogen, Waltham, MA) in Opti-MEM medium (Invitrogen, Waltham, MA). Cells were harsvested after 72 h and subjected for either real-time PCR/ WB analyses or cell viability assays in the presence of different drugs.

### Confocal microscopy

Approximately 1 × 10^6^ HEK293 cells grown 970% confluency) on 22 mm × 22 mm coverslips (Corning Inc., NY, USA) plated into each well of a six-well plate were transfected with GFP-LC3 expressing plasmid with either vector control or RFP-tagged EBNA3C expression construct using Lipofectamine 3000. After 36 h of transfection, cells were further treated with Earle’s balanced salts solution (EBSS; Invitrogen Inc., USA) to induce autophagy. Cells were then fixed and permeabilized with 4% paraformaldehyde (PFA; Sigma-Aldrich Corp. St. Louis, MO) and 0.1% Triton X-100 (Sigma-Aldrich Corp. St. Louis, MO) followed by blocking with 5% BSA (BIO-RAD, Hercules, CA) at room temperature for 1 h. Nucleus were counterstained with 4′, 6′,-diamidino-2-phenylindole (DAPI; BIO-RAD, Hercules, CA) for 30′ at room temperature. Next, cells were washed in 1× PBS for three times and mounted using an antifade mounting media (Sigma-Aldrich Corp. St. Louis, MO). The images were obtained by a Fluoview FV300 confocal microscope and subsequently analyzed by FLUOVIEW software (Olympus Inc., Melville, NY).

### Subcellular fractionation assay

Approximately 10 × 10^6^ HEK293 cells transfected with either vector or myc-EBNA3C plasmids were subjected to subcellular fractionation as per manufacturer’s protocol (BIO-RAD, Hercules, CA). Nuclear and cytoplasmic protein fractions were measured by Bradford protein assay and ~50 µg of total protein was resolved by SDS-PAGE. The efficiency of nuclear and cytoplasmic fractionation was confirmed by western blot analyses against nuclear protein Histone and cytoplasmic protein GAPDH, respectively.

### PCR microarray

To identify the transcriptional deregulation of autophagy genes, a 96-well set-up ‘Human Autophagy RT^2^ Profiler^TM^ PCR Array’ (SABiosciences, Frederick, MD) was performed using ~10 × 10^6^ BJAB and BJAB stably expressing EBNA3C cells with or without the treatment with EBSS for 3 h. Approximately 5 µg of amplified cDNA were subjected to PCR microarray analyses according to the manufacturer’s instructions. Real-time PCR was performed was performed using SsoFast EvaGreen Supermix (BIO-RAD, Hercules, CA) in CFX Connect™ real-time PCR detection system under the same conditions as described above. Data analysis is based on the 2^−ΔΔCt^ method with normalization of the raw data to the BJAB control sample (untreated) and represented as heat map analyses (log_2_). Data analysis was carried out using an algorithm provided at manufacturer’s web portal. Similar experiments were carried out for two times. Each array contained five housekeeping genes (HKGs)—*ACTB*, *B2M*, *GAPDH*, *HPRT1,* and *RPLP0*. The average ΔCt value of HKGs was used to normalize the sample data. Any Ct value greater than 35 of total 84-key genes was not accounted in calculation. If the Ct value of the human genomic DNA control (HGDC) was greater than 30, it indicated RNA was free of any genomic DNA contamination. The statistical analysis was performed based on the calculation of *p*-values using a Student’s *t*-test. The 84 ATGs of the profiler array are listed in Table S3.

### ChIP-Seq data analyses

ChIP-Seq analyses (GEO dataset IDs: GSE52632^14^ and GSE26386^36^) were carried out using PICS (probabilistic Inference for *ChIP*-seq)^69^ and MACS2 (model-based analyses of ChIP-seq)^70^ algorithms for sample replicates downloaded from corresponding SRA files against the matching input file. Fastq files were extracted from SRA files and aligned against human reference genome (Hg19) using Strand NGS with default parameters. BAM files were assessed for duplicate reads and subsequently removed. A processed non-redundant BAM file was then analysed to identify peaks using MACS2 and PICS with default parameters. For the analysis, peaks with a false discovery rate (FDR) of <1% were selected. Replicates were combined by retaining only those peaks that were present in both samples. *p*-values were calculated based on hypergeometric distribution and corrected for multiple testing using Bonferonni correction.

### Chromatin immunoprecipitation (ChIP) assay

Approximately 10 × 10^6^ BJAB stably expressing EBNA3C cells were either left untreated (DMSO control) or treated with EBSS for 6 h. Cells were then harvested and processed using a ChIP assay kit from Invitrogen (MAGnify™ Chromatin Immunoprecipitation System; Invitrogen, Thermo Fisher Scientific, Waltham, MA) essentially according to the manufacturer’s protocol. Briefly, cells were washed with 1× PBS and cross-linked with 1% formaldehyde (Sigma-Aldrich Corp. St. Louis, MO) for 10 min at room temperature. After quenching the cross-linking reaction by 125 mM glycine for 5 min at room temperature, the cells were washed with ice cold 1× PBS. Next, cell pellets were lysed in lysis buffer containing 1 × protease inhibitor cocktail, and then sonicated to attain chromatin fragments of approximately 200–500 bp using a Ultrasonic Processor UP50H (Hielscher Ultrasonics GmbH, Germany) with six sets of 10 s pulses at 50% of maximum power. After saving a 10% of the solubilzed chromatin as input, the sheared chromatin was diluted to 10-fold and subjected to immunoprecipitation using six different antibodies (1 µg each) directed against EBNA3C, H3K4me1, H3K4me3, H3K9ac, H3K27ac, and H3K27me3 along with corresponding IgG control coupled with Dynabeads for overnight at 4 °C. Antibody-chromatin complexes were pulled-down using DynaMag^TM^-PCR Magnet. After de-crosslinking and proteinase K treatment the immunoprecipitated DNA was extracted and diluted to 10-fold, quantified and subjected to qPCR analyses as described above. The primers for ChIP assay are listed in Table S9. ChiP-PCR primers were obtained from Integrated DNA Technologies, Inc. (Coralville, IA, USA).

### Statistical analysis

All the data represented are as the mean values with standard errors of means (SEM). Statistical significance of differences in the mean values was analyzed using the Student’s *t*-test two tail distribution and equal variances between two samples. *p*-value below 0.05 was considered as significant.

## Electronic supplementary material

Figure S1

Figure S2

Figure S3

Figure S4

Figure S5

Figure S6

Figure S7

Figure S8

Table S1

Table S2

Table S3

Table S4

Table S5

Table S6

Table S7

Table S8

Supplementary figure legends
