# Supplementary figures and images for: Transcriptional and epigenetic modulation of autophagy promotes EBV oncoprotein EBNA3C induced B-cell survival
Source: Cell Death Dis. 2018 May 22;9(6):605. doi: 10.1038/s41419-018-0668-9 (PMC5964191; doi:10.1038/s41419-018-0668-9)

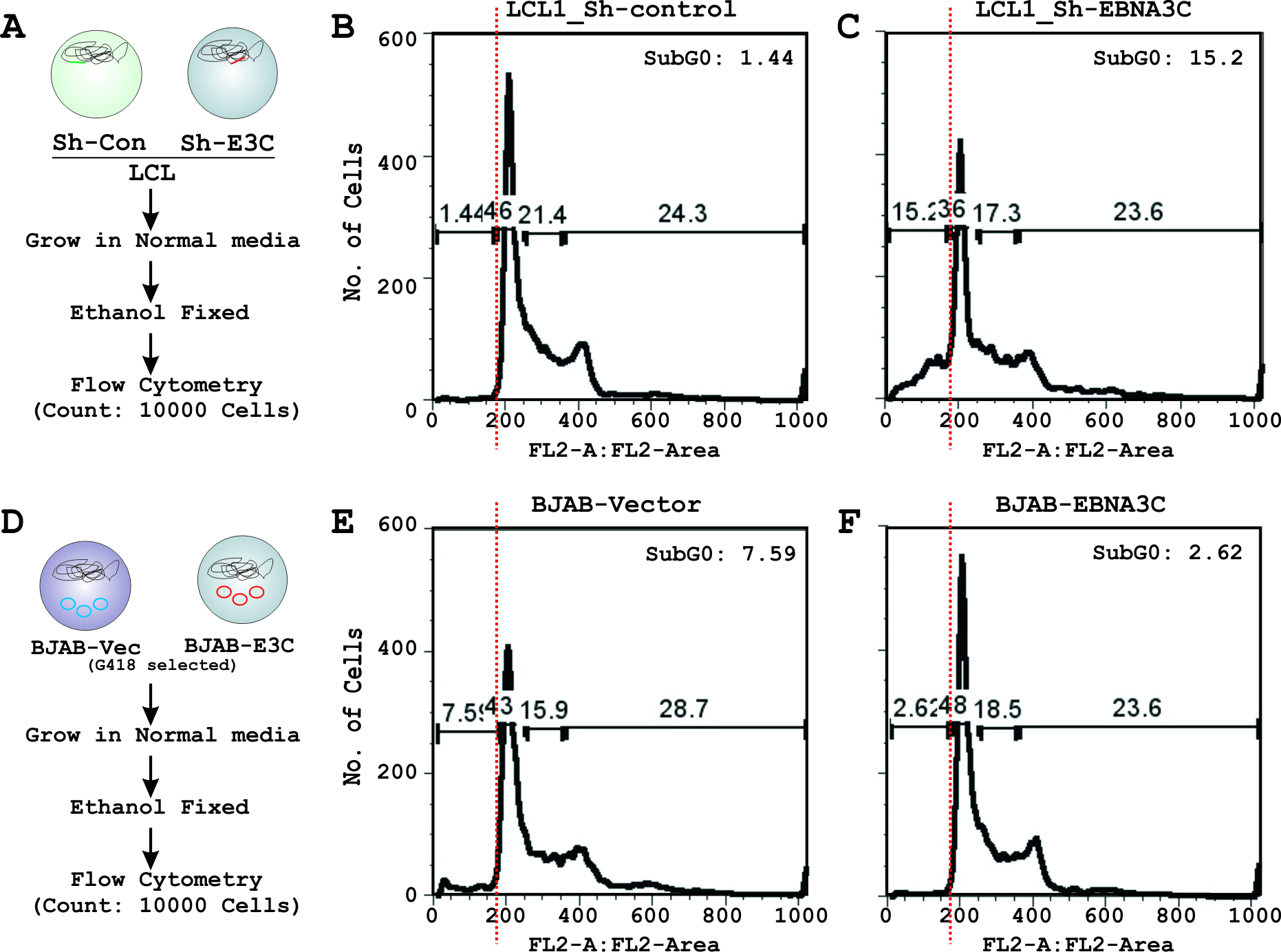

Supplement: Supplementary file 1 — Figure S1 [file 41419_2018_668_MOESM1_ESM.tif]

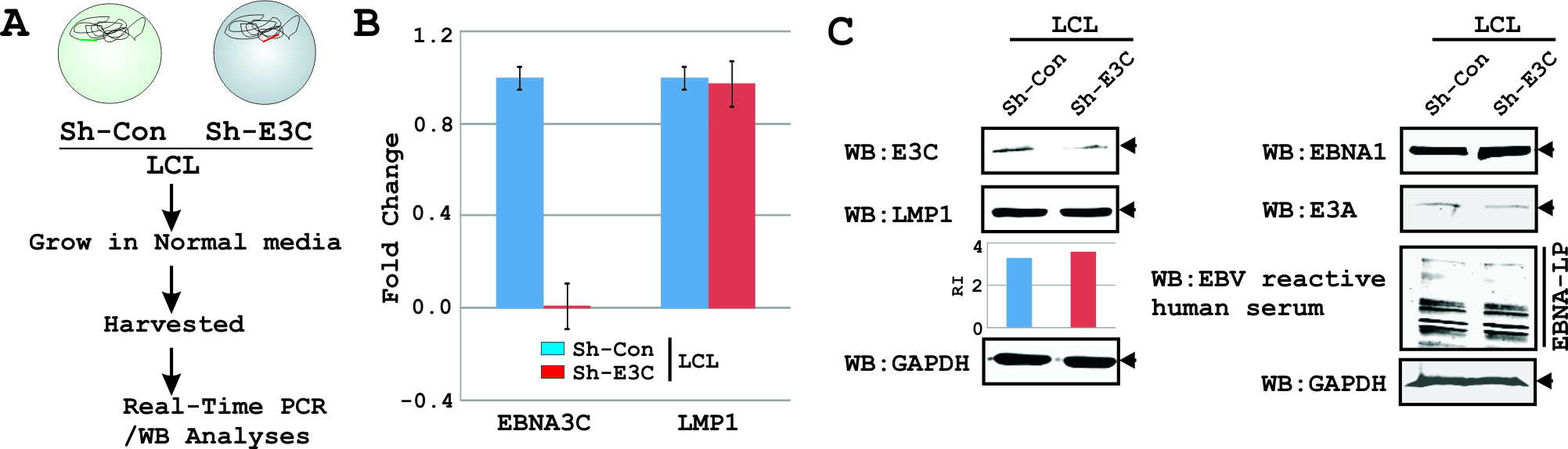

Supplement: Supplementary file 2 — Figure S2 [file 41419_2018_668_MOESM2_ESM.tif]

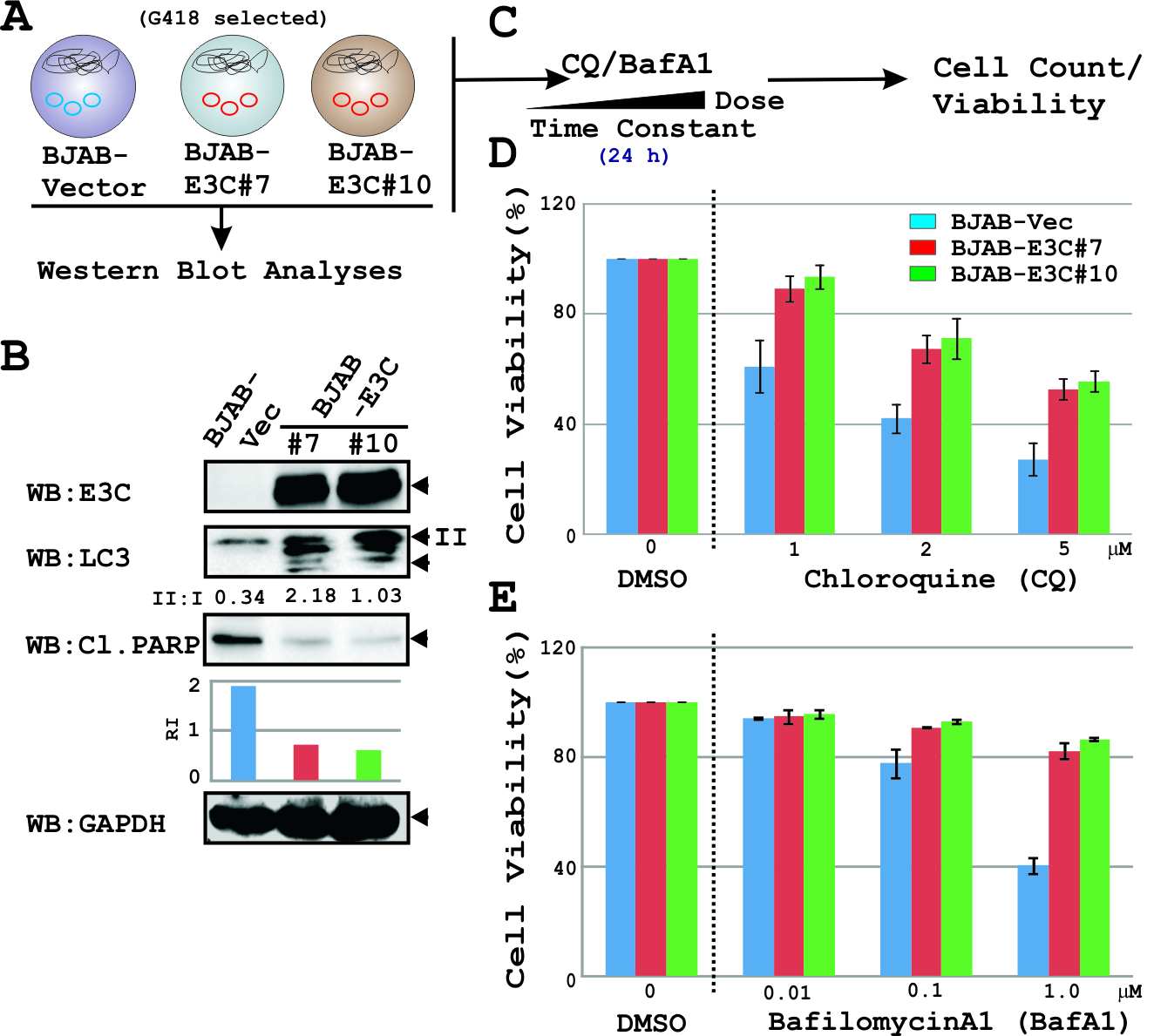

Supplement: Supplementary file 3 — Figure S3 [file 41419_2018_668_MOESM3_ESM.tif]

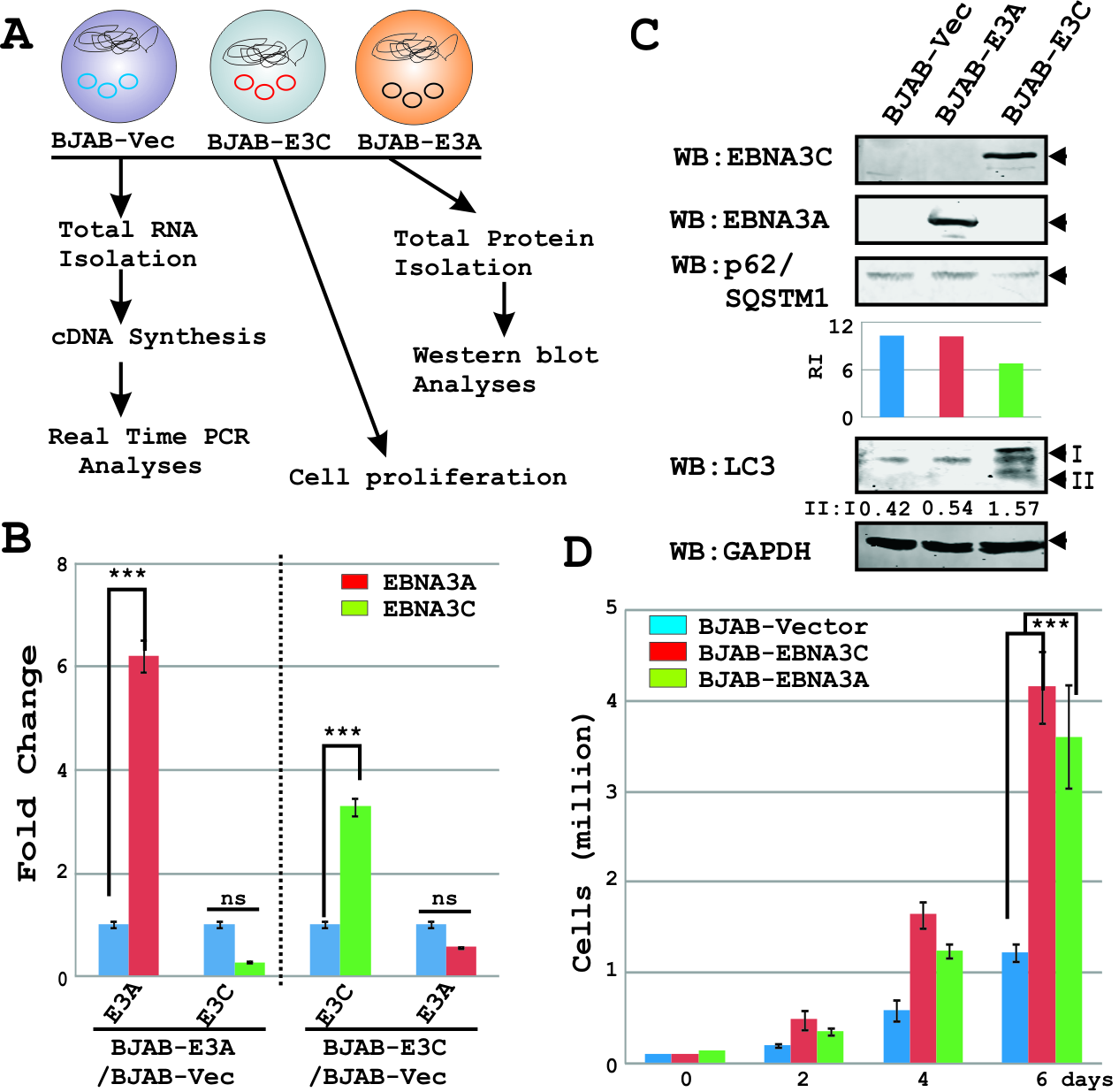

Supplement: Supplementary file 4 — Figure S4 [file 41419_2018_668_MOESM4_ESM.tif]

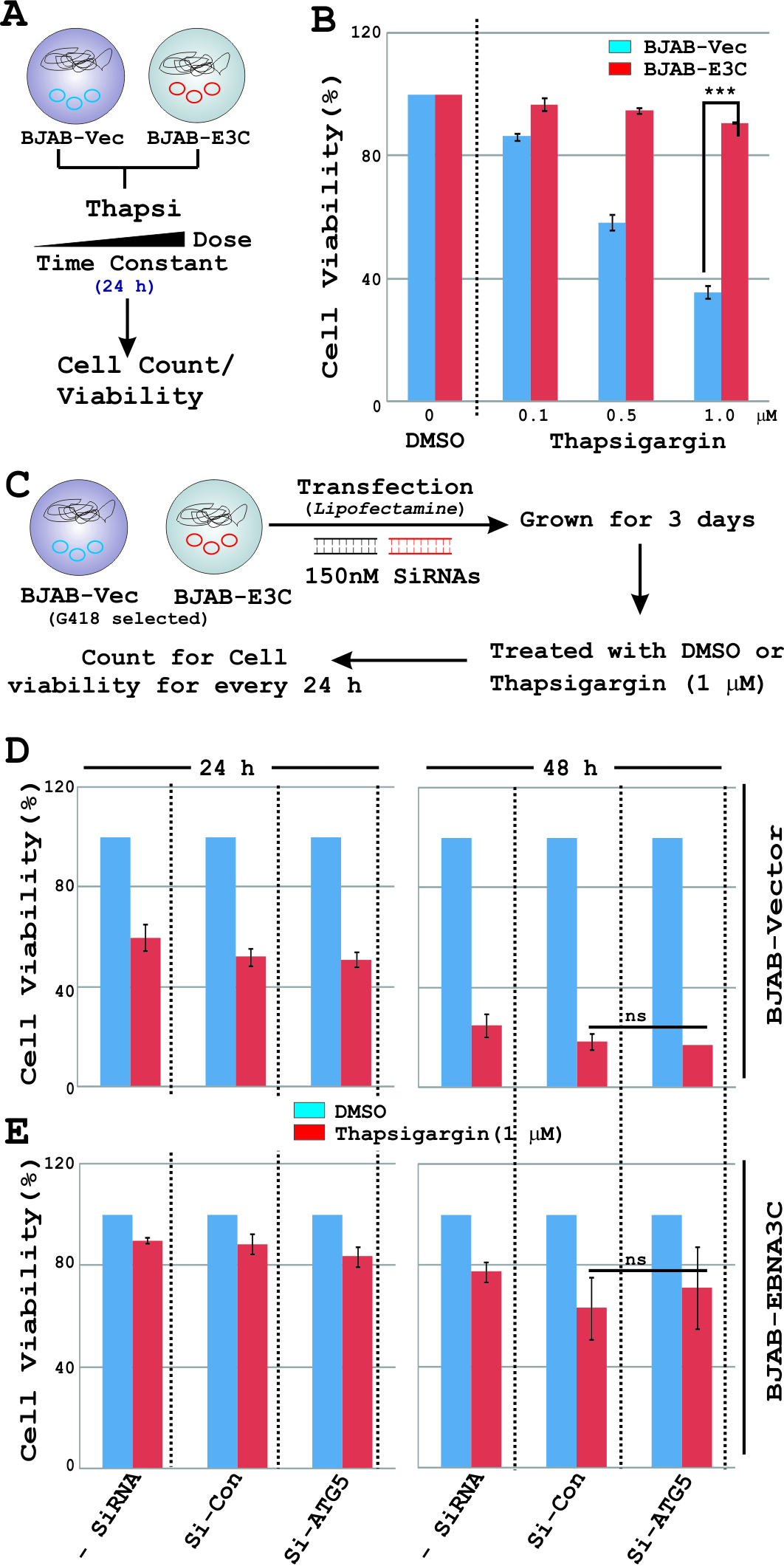

Supplement: Supplementary file 5 — Figure S5 [file 41419_2018_668_MOESM5_ESM.tif]

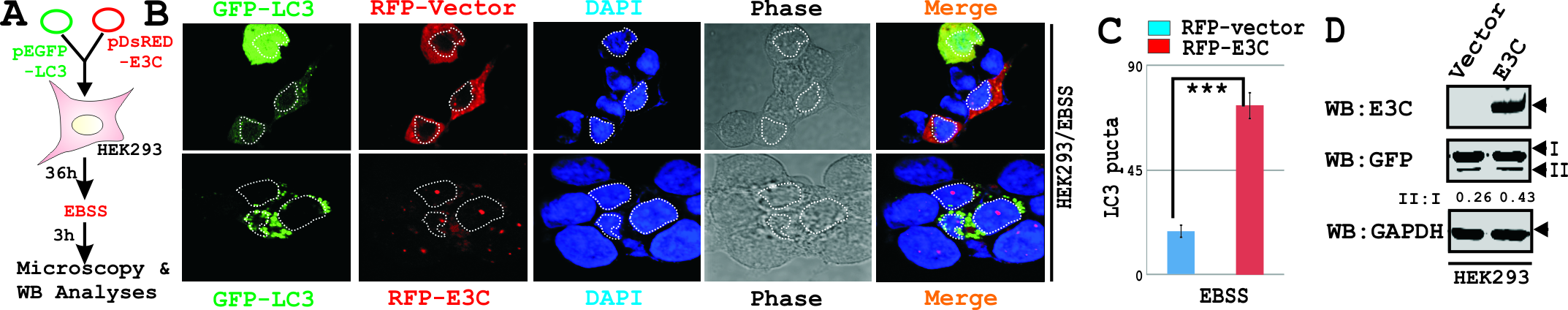

Supplement: Supplementary file 6 — Figure S6 [file 41419_2018_668_MOESM6_ESM.tif]

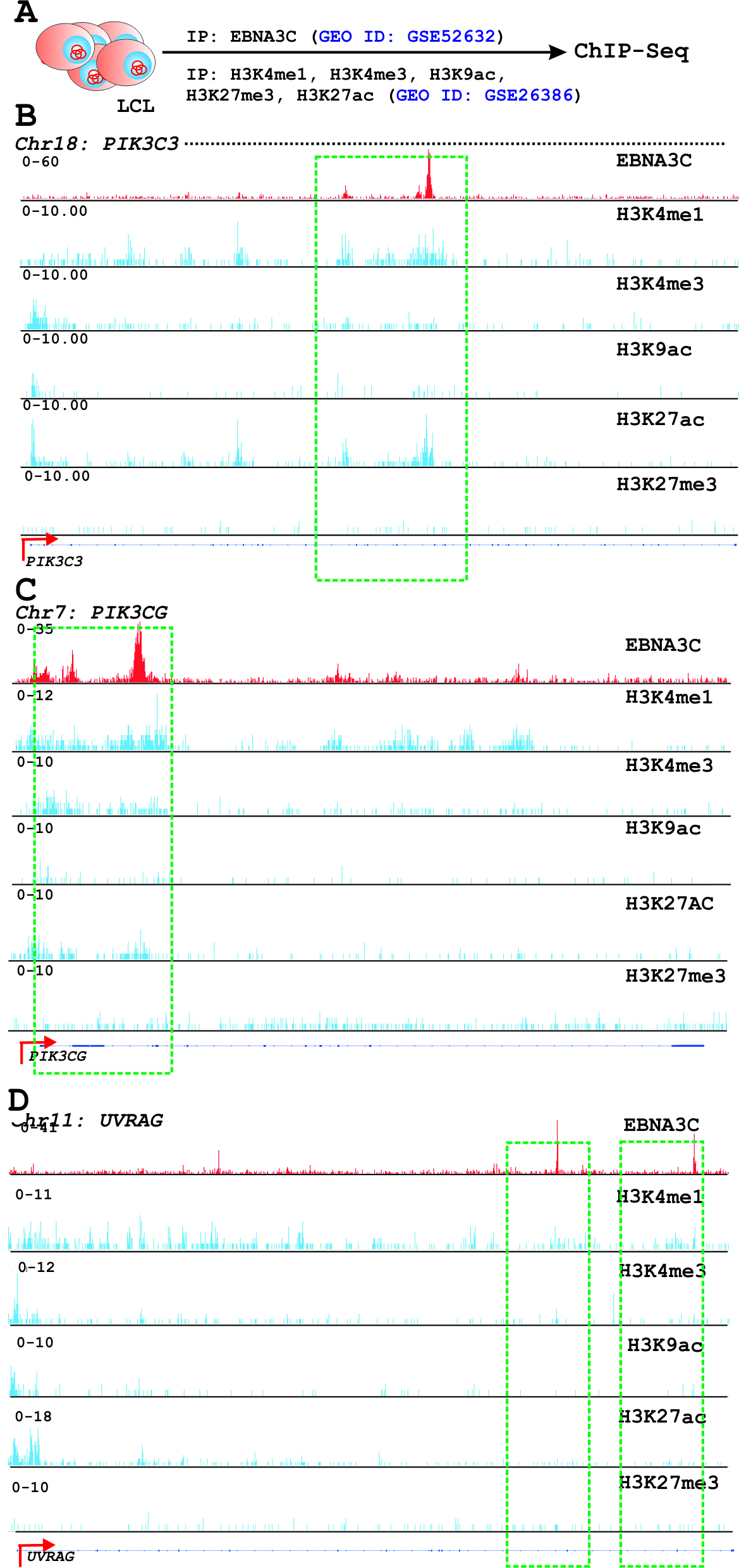

Supplement: Supplementary file 7 — Figure S7 [file 41419_2018_668_MOESM7_ESM.tif]

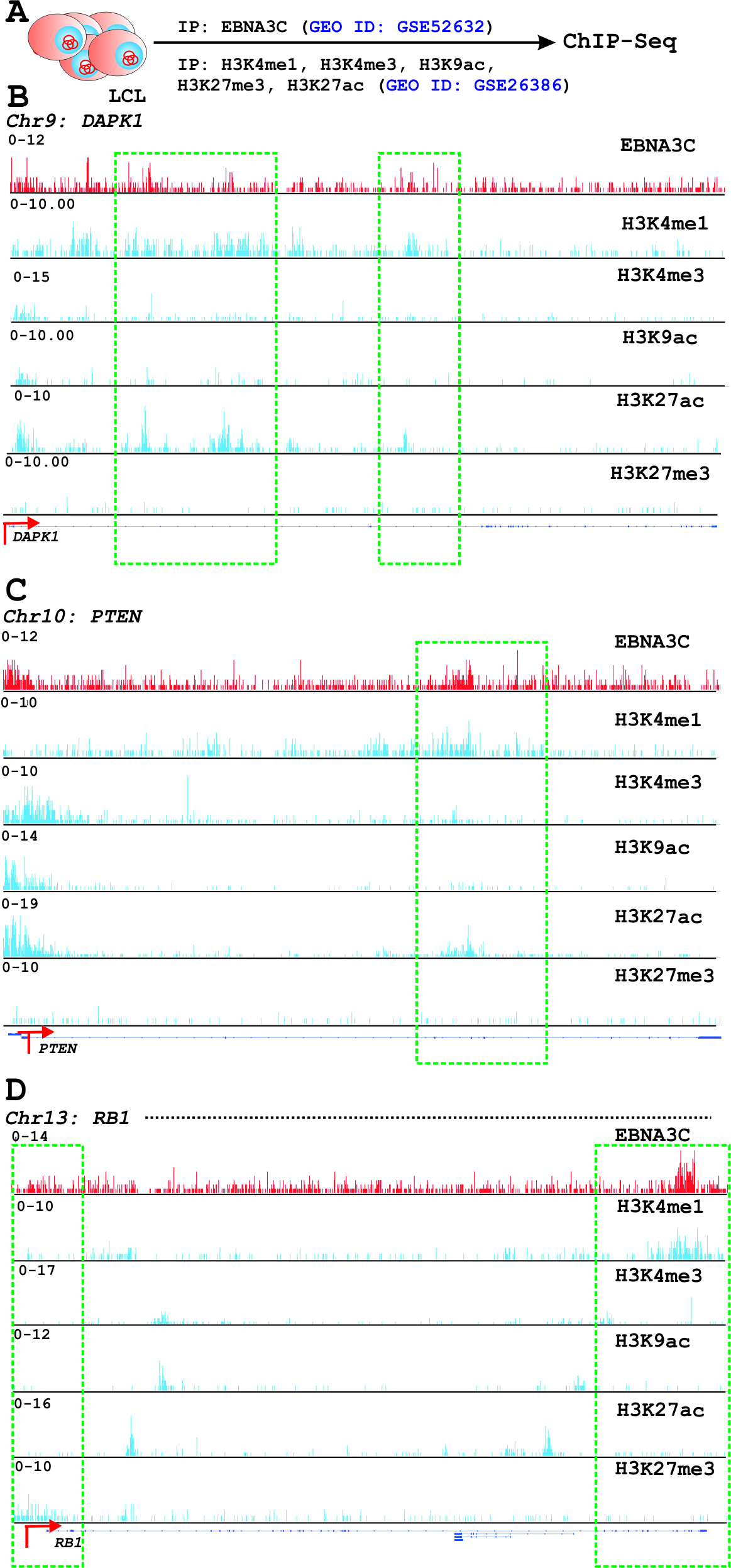

Supplement: Supplementary file 8 — Figure S8 [file 41419_2018_668_MOESM8_ESM.tif]
