# Supplementary material for: Transcriptional and epigenetic modulation of autophagy promotes EBV oncoprotein EBNA3C induced B-cell survival
Source: Cell Death Dis. 2018 May 22;9(6):605. doi: 10.1038/s41419-018-0668-9 (PMC5964191; doi:10.1038/s41419-018-0668-9)
Supplement: Supplementary file 9 — Table S1 [file 41419_2018_668_MOESM9_ESM.docx]

**Table S1.** **Real time PCR primers.**

| **SL No.** | **Gene Name** | **Reference Sequences** | **Primer Sequences** |
| --- | --- | --- | --- |
|  | LMP1 | YP_401722.1 | Fw- 5’-CCCGCACCCTCAACAAGCTACCGAT-3’  Rv- 5’-TTGTCAGGACCACCTCCAGGTGCGC-3’ |
|  | EBNA3A | YP_401669.1 | Fw- 5’-GCCCTGGATGACAACATGGA-3’  Rv- 5’-CAGGTGGGCATCTTCTGCTT-3’ |
|  | EBNA3C | YP_401671.1 | Fw- 5’-AAGGTGCATTTACCCCACTG-3’  Rv- 5’-AGCAGTAGCTTGGGAACACC-3’ |
|  | ATG3 | NM_022488 | Fw- 5’- CATGTTGGACAGTGGTGGAC-3’  Rv- 5’-GCACTGGAAGTGGCTGAGTA-3’ |
|  | ATG5 | NM_004849 | Fw- 5’-CAGGACGAAACAGCTTCTGA-3’  Rv- 5’-TCAATCGGAAACTCATGGAA-3’ |
|  | ATG7 | NM_006395 | Fw- 5’-GATGGAGAGCTCCTCAGCA-3’  Rv- 5’-ATTGCTGCATCAAGAAACCC-3’ |
|  | ATG12 | NM_004707 | Fw- 5’- TTCCAACTTCTTGGTCTGGG-3’  Rv- 5’- CGAACCATCCAAGGACTCAT-3’ |
|  | ATG 16L1 | NM_017974 | Fw- 5’- TCGTTTCTGGGACATTCGAT-3’  Rv- 5’-TTTGGGTCTTTCCTGACTCGA-3’ |
|  | BECN1 | NM_003766 | Fw- 5’-CTCCTGGGTCTCTCCTGGTT-3  Rv- 5’-TGGACACGAGTTTCAAGATCC-3’ |
|  | SQSTM1 | NM_003900 | Fw- 5’-TTCTTTTCCCTCCGTGCTC-3’  Rv- 5’-GGATCCGAGTGTGAATTTCC-3’ |
|  | MAP1LC3A | NM_181509 | Fw- 5’-CCTTGTAGCGCTCGATGAT-3’  Rv- 5’-CATGTGGAAAAGCAGCTGTG-3’ |
|  | MAP1LC3B | NM_022818 | Fw- 5’-AAGCTGCTTCTCACCCTTGT-3’  Rv- 5’-GAGAAGACCTTCAAGCAGCG-3’ |
|  | CDKN1B | NM_004064 | Fw- 5’- CGTCAAACGTAAACAGCTCG-3’  Rv- 5’-CATTCCATGAAGTCAGCGAT-3’ |
|  | CDKN2A | NM_000077 | Fw- 5’- GGTCGGGTGAGAGTGGC-3’  Rv- 5’- CCCAACGCACCGAATAGTTA-3’ |
