# Supplementary material for: Transcriptional and epigenetic modulation of autophagy promotes EBV oncoprotein EBNA3C induced B-cell survival
Source: Cell Death Dis. 2018 May 22;9(6):605. doi: 10.1038/s41419-018-0668-9 (PMC5964191; doi:10.1038/s41419-018-0668-9)
Supplement: Supplementary file 10 — Table S2 [file 41419_2018_668_MOESM10_ESM.docx]

**Table S3.** **Description of autophagy genes in PCR-microarray.**

| **Sl No.** | **Gene Name** | **Description** |
| --- | --- | --- |
| 1. | AKT1 | V-akt murine thymoma viral oncogene homolog 1 |
| 2. | AMBRA1 | Autophagy/beclin-1 regulator 1 |
| 3. | APP | Amyloid beta (A4) precursor protein |
| 4. | ATG10 | ATG10 autophagy related 10 homolog (S. cerevisiae) |
| 5. | ATG12 | ATG12 autophagy related 12 homolog (S. cerevisiae) |
| 6. | ATG16L1 | ATG16 autophagy related 16-like 1 (S. cerevisiae) |
| 7. | ATG16L2 | ATG16 autophagy related 16-like 2 (S. cerevisiae) |
| 8. | ATG3 | ATG3 autophagy related 3 homolog (S. cerevisiae) |
| 9. | ATG4A | ATG4 autophagy related 4 homolog A (S. cerevisiae) |
| 10. | ATG4B | ATG4 autophagy related 4 homolog B (S. cerevisiae) |
| 11. | ATG4C | ATG4 autophagy related 4 homolog C (S. cerevisiae) |
| 12. | ATG4D | ATG4 autophagy related 4 homolog D (S. cerevisiae) |
| 13. | ATG5 | ATG5 autophagy related 5 homolog (S. cerevisiae) |
| 14. | ATG7 | ATG7 autophagy related 7 homolog (S. cerevisiae) |
| 15. | ATG9A | ATG9 autophagy related 9 homolog A (S. cerevisiae) |
| 16. | ATG9B | ATG9 autophagy related 9 homolog B (S. cerevisiae) |
| 17. | BAD | BCL2-associated agonist of cell death |
| 18. | BAK1 | BCL2-antagonist/killer 1 |
| 19. | BAX | BCL2-associated X protein |
| 20. | BCL2 | B-cell CLL/lymphoma 2 |
| 21. | BCL2L1 | BCL2-like 1 |
| 22. | BECN1 | Beclin 1, autophagy related |
| 23. | BID | BH3 interacting domain death agonist |
| 24. | BNIP3 | BCL2/adenovirus E1B 19kDa interacting protein 3 |
| 25. | CASP3 | Caspase 3, apoptosis-related cysteine peptidase |
| 26. | CASP8 | Caspase 8, apoptosis-related cysteine peptidase |
| 27. | CDKN1B | Cyclin-dependent kinase inhibitor 1B (p27, Kip1) |
| 28. | CDKN2A | Cyclin-dependent kinase inhibitor 2A (melanoma, p16, inhibits CDK4) |
| 29. | CLN3 | Ceroid-lipofuscinosis, neuronal 3 |
| 30. | CTSB | Cathepsin B |
| 31. | CTSD | Cathepsin D |
| 32. | CTSS | Cathepsin S |
| 33. | CXCR4 | Chemokine (C-X-C motif) receptor 4 |
| 34. | DAPK1 | Death-associated protein kinase 1 |
| 35. | DRAM1 | DNA-damage regulated autophagy modulator 1 |
| 36. | DRAM2 | DNA-damage regulated autophagy modulator 2 |
| 37. | EIF2AK3 | Eukaryotic translation initiation factor 2-alpha kinase 3 |
| 38. | EIF4G1 | Eukaryotic translation initiation factor 4 gamma, 1 |
| 39. | ESR1 | Estrogen receptor 1 |
| 40. | FADD | Fas (TNFRSF6)-associated via death domain |
| 41. | FAS | Fas (TNF receptor superfamily, member 6) |
| 42. | GAA | Glucosidase, alpha; acid |
| 43. | GABARAP | GABA(A) receptor-associated protein |
| 44. | GABARAPL1 | GABA(A) receptor-associated protein like 1 |
| 45. | GABARAPL2 | GABA(A) receptor-associated protein-like 2 |
| 46. | HDAC1 | Histone deacetylase 1 |
| 47. | HDAC6 | Histone deacetylase 6 |
| 48. | HGS | Hepatocyte growth factor-regulated tyrosine kinase substrate |
| 49. | HSP90AA1 | Heat shock protein 90kDa alpha (cytosolic), class A member 1 |
| 50. | HSPA8 | Heat shock 70kDa protein 8 |
| 51. | HTT | Huntingtin |
| 52. | IFNG | Interferon, gamma |
| 53. | IGF1 | Insulin-like growth factor 1 (somatomedin C) |
| 54. | INS | Insulin |
| 55. | IRGM | Immunity-related GTPase family, M |
| 56. | LAMP1 | Lysosomal-associated membrane protein 1 |
| 57. | MAP1LC3A | Microtubule-associated protein 1 light chain 3 alpha |
| 58. | MAP1LC3B | Microtubule-associated protein 1 light chain 3 beta |
| 59. | MAPK14 | Mitogen-activated protein kinase 14 |
| 60. | MAPK8 | Mitogen-activated protein kinase 8 |
| 61. | MTOR | Mechanistic target of rapamycin (serine/threonine kinase) |
| 62. | NFKB1 | Nuclear factor of kappa light polypeptide gene enhancer in B-cells 1 |
| 63. | NPC1 | Niemann-Pick disease, type C1 |
| 64. | PIK3C3 | Phosphoinositide-3-kinase, class 3 |
| 65. | PIK3CG | Phosphoinositide-3-kinase, catalytic, gamma polypeptide |
| 66. | PIK3R4 | Phosphoinositide-3-kinase, regulatory subunit 4 |
| 67. | PRKAA1 | Protein kinase, AMP-activated, alpha 1 catalytic subunit |
| 68. | PTEN | Phosphatase and tensin homolog |
| 69. | RAB24 | RAB24, member RAS oncogene family |
| 70. | RB1 | Retinoblastoma 1 |
| 71. | RGS19 | Regulator of G-protein signaling 19 |
| 72. | RPS6KB1 | Ribosomal protein S6 kinase, 70kDa, polypeptide 1 |
| 73. | SNCA | Synuclein, alpha (non A4 component of amyloid precursor) |
| 74. | SQSTM1 | Sequestosome 1 |
| 75. | TGFB1 | Transforming growth factor, beta 1 |
| 76. | TGM2 | Transglutaminase 2 (C polypeptide, protein-glutamine-gamma-glutamyltransferase) |
| 77. | TMEM74 | Transmembrane protein 74 |
| 78. | TNF | Tumor necrosis factor |
| 79. | TNFSF10 | Tumor necrosis factor (ligand) superfamily, member 10 |
| 80. | TP53 | Tumor protein p53 |
| 81. | ULK1 | Unc-51-like kinase 1 (C. elegans) |
| 82. | ULK2 | Unc-51-like kinase 2 (C. elegans) |
| 83. | UVRAG | UV radiation resistance associated gene |
| 84. | WIPI1 | WD repeat domain, phosphoinositide interacting 1 |
