# Supplementary material for: Transcriptional and epigenetic modulation of autophagy promotes EBV oncoprotein EBNA3C induced B-cell survival
Source: Cell Death Dis. 2018 May 22;9(6):605. doi: 10.1038/s41419-018-0668-9 (PMC5964191; doi:10.1038/s41419-018-0668-9)
Supplement: Supplementary file 16 — Table S8 [file 41419_2018_668_MOESM16_ESM.docx]

**Table S8. ChIP-PCR primers for selected genes.**

| **SL No.** | **Genes** | **Primer Location** | **Primer Sequences** |
| --- | --- | --- | --- |
|  | *ATG3* | 112280960-112281160 | Fw-5’-TGCAATAGGCGGCTTAGAGG-3’  Rv-5'-GACGCAGGCGCAATTCCTT-3’ |
|  |  | 112281036-112281236 | Fw-5’-TGCTACTACGGGGCTAGACA-3’  Rv-5'-TCACAGTCAGGGACTACCCTT-3’ |
|  | *ATG5* | 106668456-106668680 | Fw-5’-TACCCAAAAGAAGGAACCACTACC-3’  Rv-5'-CATGCCAGAATACACAACACCTG-3’ |
|  |  | 106654928-106655072 | Fw-5’-AGTCACCTTTGAAACTGTCCTTG-3’  Rv-5'-CATGCCAGAATACACAACACCTG-3’ |
|  | *ATG7* | 11330700-11330900 | Fw-5’-TGAAACACACTTACATTGATGCCT-3’  Rv-5'-CCCTGGACAGTGCAAGTGAA-3’ |
|  |  | 11342539-11342842 | Fw-5’-TTCTTTCCTTGACTGTGTCTGAAT-3’  Rv-5'-ACCCTGGACAGTGCAAGTG-3’ |
|  | *DRAM1* | 102271192-102271288 | Fw-5’-GCCGAGTGTCCAAACCAAAAG-3’  Rv-5'-TCCAAGCGGACGCGACTA-3’ |
|  |  | 102285408-102285512 | Fw-5’-GCACAACCTACCCCTAAAATAACTG-3’  Rv-5'-AAAGAGGACAAGGCCAGCAAA-3’ |
|  | *CDKN1B* | 12867263-12867367 | Fw-5’-TCGCAGAAACATTTGGGGCT-3’  Rv-5'-TTCAGATGGGGTGAATGAGCA-3’ |
|  |  | 12868257-12869139 | Fw-5’-CCAGTGAGAACCATTAGGAAGGA-3’  Rv-5'-CATTCGGTTGGTACCGGAGT-3’ |
|  | *CDKN2A* | 21995880-21996008 | Fw-5’-TGGGTGTGTGCCAGAGGATTC-3’  Rv-5'-ACTGAGAAGCGGGCCACAT-3’ |
|  |  | 21986080-21986472 | Fw-5’-GTGGTTCCTGTGCTGGTGAAT-3’  Rv-5'-TTAACCCCTTTGGGCCTCTAC-3’ |
