# Supplementary material for: Transcriptional and epigenetic modulation of autophagy promotes EBV oncoprotein EBNA3C induced B-cell survival
Source: Cell Death Dis. 2018 May 22;9(6):605. doi: 10.1038/s41419-018-0668-9 (PMC5964191; doi:10.1038/s41419-018-0668-9)
Supplement: Supplementary file 17 — Supplementary figure legends [file 41419_2018_668_MOESM17_ESM.docx]

## Supporting Information

**Figure S1. EBNA3C inhibits apoptosis.** ~1 x 10^6^ (A-C) BJAB-vector control and BJAB stably expressing EBNA3C or (D-E) LCLs stably expressing sh-control and sh-EBNA3C cells were harvested, stained with propidium iodide and subjected for flow cytometry analyses to measure SubG0 value in each panel. Representative pictures of two independent experiments are shown.

**Figure S2. EBNA3C knockdown in LCLs does not alter LMP1 expression.** (A-C) ∼10 x 10^6^ LCLs stably expressing either control sh-RNA or sh-RNA directed against EBNA3C ORF were harvested and subjected for either (B) real-time PCR or (C) WB analyses for the selected viral gene expressions. The relative changes in transcripts using the 2^−ΔΔCt^ method were represented as bar diagram in comparison to LCLs expressing control sh-RNA. Two independent experiments were carried out in similar settings and results represent as an average value for each transcript. GAPDH was used as control for both real-time PCR and WB analyses.

**Figure S3. Two independent EBNA3C stably expressing BJAB clones upregulate autophagy and rescue cell-death induced by autophagy inhibitors** (A-B) ~10 x 10^6^ BJAB cells either expressing vector control or EBNA3C (clones #7 and #10) were harvested and subjected for (B) WB analyses for the indicated antibodies. GAPDH blot was performed as loading control. Individual protein bands were quantified (pixels) by Odyssey imager software and represented as bar diagram (PARP cleavage) or at is indicated at the bottom of each corresponding lane (ratio of LC3-II/I). Representative WB pictures are shown of two independent experiments. (C-E) ~0.5 x 10^5^ cells were treated with increasing concentrations of (D) chloroquine (0 – 5 µM) and (E) bafilomycin (0 – 1 µM). (D-E) 24 h post-treatment, cell viability was checked using Trypan blue exclusion method in an automated cell counter. Error bars represent standard deviations of duplicate assays of two independent experiments. *** indicates P < 0.05.

**Figure S4. EBNA3C but not EBNA3A in BJAB stable cell lines increases basal level of autophagy.** (A) ∼10 x 10^6^ BJAB cells stably expressing vector control, EBNA3A or EBNA3C were harvested and subjected for (B) real-time PCR, (C) western blot (WB) analyses and (D) cell proliferation assays. (B) The relative changes in transcripts using the 2^−ΔΔCt^ method were represented as bar diagram in comparison to BJAB control cells. An average value with SD of two independent experiments of each transcript is presented in the bar diagram. GAPDH was used as a housekeeping gene. (C) In WB analyses GAPDH blot was performed as loading control. Individual protein bands were quantified (pixels) by Odyssey imager software and represented as bar diagram (p62 expression) or indicated at the bottom of each corresponding lane (ratio of LC3-II/I). Representative WB pictures are shown of two independent experiments. (D) As described in Fig. 1, for proliferation assay ~0.1 x 10^6^ cells plated into each well of a 6-well plate were grown for 6-days in complete RPMI and counted viable cells (million/ mL) using Trypan Blue exclusion method in an automated cell counter. Average of two independent experiments is shown as bar diagram. *** P<0.5.

**Figure S5. ATG5 knockdown does not sensitize cell-death of EBNA3C expressing B-cells in response to thapsigargin treatment.** (A-B) ~0.5 x 10^5^ BJAB-vector and BJAB stably expressing EBNA3C cells were treated with increasing doses of thapsigargin (0 – 1 µM). 24 h post-incubation cells were subjected to cell viability assay using Trypan blue exclusion method in an automated cell counter as described in Fig. 2. (C-E) As described in Fig. 3, ∼1 x 10^6^ BJAB and BJAB stably expressing EBNA3C cells (clone #10) were transfected using control and *ATG5* si-RNAs. ~0.5 x 10^5^ non-transfected or 72 h post-transfected cells were either left untreated or incubated with DMSO or 1 μM Thapsigargin for 2 days. After every 24 h viable cells were counted using Trypan Blue exclusion method in an automated cell counter. Average of two independent experiments is represented as bar diagram. *** indicates P < 0.05.

**Figure S6. Confocal and WB analyses of EBNA3C mediated autophagy upregulation under growth limiting conditions.** (A-D) ~1 x 10^6^ HEK293 cells were co-transfected with indicated plasmid constructs. 36 h post-tranfections, cells either left untreated or incubated with EBSS for 3 h were subjected for (B-C) confocal and (D) WB analyses as described in Fig. 4. (B) All panels are representative pictures and (C) the bar diagram represents the mean value of GFP-LC3 puncta from ~50 cells of 5 different fields of two independent experiments. *** indicates P < 0.05. (D) GAPDH blot was performed as loading control. LC3-II and LC3I protein band intensities (pixels) were quantified by Odyssey imager software and the ratio of LC3II/I is indicated at the bottom of each corresponding lane. *** indicates P < 0.05.

**Figure S7. Distribution of EBNA3C binding and different histone modification sites around *PIK3C3*, *PIK3CG* and *UVRAG* gene loci.** (A-D) As described in Fig. 6, ChIP-seq data were reanalyzed and displayed using IGV software similarly for (B) *PIK3C3*, (C) *PIK3CG*, (D) *UVRAG* gene loci.

**Figure S8. Distribution of EBNA3C binding and different histone modification sites around *DAPK1*, *PTEN* and *RB1* gene loci.** (A-D) As described in Fig. 6, ChIP-seq data were reanalyzed and displayed using IGV software similarly for (B) *DAPK1*, (C) *PTEN*, (D) *RB1* gene loci.

**Table S1. Real-time PCR primers.** Real-time PCR primers for checking expression of viral genes (*EBNA3A*, *EBNA3C* and *LMP1*) were synthesized based on the sequence as previously described [86]. Reference Sequence ID (RefSeq ID) of each viral gene is mentioned. Real-time PCR primers for selected genes in PCR-microarray platform were designed using a real-time PCR primer database, qPrimerDepot (https://primerdepot.nci.nih.gov/). All primers were selected at annealing temperature of ~60^0^C. RefSeq ID of each cellular gene is mentioned.

**Table S2. Description of autophagy genes in PCR-microarray.** Description of each gene in PCR-microarray platform. Source: http://www.genecards.org/.

**Table S3. EBNA3C ChIP-seq data for 84 genes from PCR-microarray platform.** EBNA3C ChIP-seq data (GEO dataset ID: GSE52632) for 84 key autophagy related genes (as described in Table S2) from PCR-microarray platform using PICS and MACS2 algorithms.

**Table S4. H3K4me1 ChIP-seq data for 84 genes from PCR-microarray platform.** H3K4me1 ChIP-seq data (GEO dataset ID: GSE26386) for 84 key autophagy related genes (as described in Table S2) from PCR-microarray platform using PICS and MACS2 algorithms.

**Table S5. H3K4me3 ChIP-seq data for 84 genes from PCR-microarray platform.** H3K4me3 ChIP-seq data for 84 key autophagy related genes (as described in Table S2) from PCR-microarray platform using PICS and MACS2 algorithms.

**Table S6. H3K9ac ChIP-seq data for 84 genes from PCR-microarray platform.** H3K9ac ChIP-seq data for 84 key autophagy related genes (as described in Table S2) from PCR-microarray platform using PICS and MACS2 algorithms.

**Table S7. H3K27ac ChIP-seq data for 84 genes from PCR-microarray platform.** H3K27ac ChIP-seq data for 84 key autophagy related genes (as described in Table S2) from PCR-microarray platform using PICS and MACS2 algorithms.

**Table S8. List of ChIP-PCR primers.** Sequence of ChIP-PCR oligos of EBNA3C bound regions of the selected autophagy related gene loci. Binding sequences were determined using UCSC Genome Browser Gateway (https://genome.ucsc.edu/cgi-bin/hgGateway) form most consistent EBNA3C binding regions/peaks in both replicates considering Hg19 as reference genome. ChIP primers were designed by NCBI primer BLAST application (https://www.ncbi.nlm.nih.gov/tools/primer-blast/). The optimum annealing temperature for all the primers is ~60^0^C.
